# Heptad stereotypy, S/Q layering, and remote origin of the SARS-CoV-2 fusion core

**DOI:** 10.1093/ve/veab097

**Published:** 2021-12-15

**Authors:** Chiara Marchetti, Serena Vaglietti, Francesca Rizzo, Giovanna Di Nardo, Luca Colnaghi, Mirella Ghirardi, Ferdinando Fiumara

**Affiliations:** Rita Levi Montalcini Department of Neuroscience, University of Torino, Corso Raffaello 30, Torino 10125, Italy; Rita Levi Montalcini Department of Neuroscience, University of Torino, Corso Raffaello 30, Torino 10125, Italy; Istituto Zooprofilattico Sperimentale (IZS) del Piemonte, Liguria e Valle d’Aosta, Via Bologna 148, Torino 10148, Italy; Department of Life Sciences and Systems Biology (DBIOS), University of Torino, Via Accademia Albertina 13, Torino 10123, Italy; Division of Neuroscience, IRCCS San Raffaele Scientific Institute, Via Olgettina 60, Milano 20132, Italy; School of Medicine, Vita-Salute San Raffaele University, Via Olgettina 58, Milano 20132, Italy; Rita Levi Montalcini Department of Neuroscience, University of Torino, Corso Raffaello 30, Torino 10125, Italy; National Institute of Neuroscience (INN), University of Torino, Corso Raffaello 30, Torino 10125, Italy; Rita Levi Montalcini Department of Neuroscience, University of Torino, Corso Raffaello 30, Torino 10125, Italy; National Institute of Neuroscience (INN), University of Torino, Corso Raffaello 30, Torino 10125, Italy

**Keywords:** SARS-CoV-2 spike protein, Covid-19, coiled-coil 6-helix bundle fusion core, heptad stereotypy, serine-rich, SARS-CoV-2 origin

## Abstract

The fusion of the SARS-CoV-2 virus with cells, a key event in the pathogenesis of Covid-19, depends on the assembly of a six-helix fusion core (FC) formed by portions of the spike protein heptad repeats (HRs) 1 and 2. Despite the critical role in regulating infectivity, its distinctive features, origin, and evolution are scarcely understood. Thus, we undertook a structure-guided positional and compositional analysis of the SARS-CoV-2 FC, in comparison with FCs of related viruses, tracing its origin and ongoing evolution. We found that clustered amino acid substitutions within HR1, distinguishing SARS-CoV-2 from SARS-CoV-1, enhance local heptad stereotypy and increase sharply the FC serine-to-glutamine (S/Q) ratio, determining a neat alternate layering of S-rich and Q-rich subdomains along the post-fusion structure. Strikingly, SARS-CoV-2 ranks among viruses with the highest FC S/Q ratio, together with highly syncytiogenic respiratory pathogens (RSV, NDV), whereas MERS-Cov, HIV, and Ebola viruses display low ratios, and this feature reflects onto S/Q segregation and H-bonding patterns. Our evolutionary analyses revealed that the SARS-CoV-2 FC occurs in other SARS-CoV-1-like Sarbecoviruses identified since 2005 in Hong Kong and adjacent regions, tracing its origin to >50 years ago with a recombination-driven spread. Finally, current mutational trends show that the FC is varying especially in the FC1 evolutionary hotspot. These findings establish a novel analytical framework illuminating the sequence/structure evolution of the SARS-CoV-2 FC, tracing its long history within Sarbecoviruses, and may help rationalize the evolution of the fusion machinery in emerging pathogens and the design of novel therapeutic fusion inhibitors.

## 1. Introduction

The recent emergence of the SARS-CoV-2, a zoonotic coronavirus (CoV) causing severe respiratory and systemic disease in humans (‘Covid-19’; Wu et al. 2020), poses the need to better understand its biological features and evolution for therapeutic and public health purposes.

SARS-CoV-2 belongs to the Sarbecovirus CoV subgenus together with SARS CoV-1 which is the most closely related human CoV. Host tropism and infectivity of SARS-CoV-2 depend on a ‘spike’ protein mediating fusion with target cells upon receptor binding (Whittaker, Daniel, and Millet 2021). The S gene encoding this protein is a preferential site for recombination which can lead to rapid changes in virus tropism, infectivity, and antigenicity by reshuffling key functional regions of the spike protein (Bobay, O’Donnell, and Ochman 2020). Among these regions, the fusion core (FC), which is formed by parts of the heptad repeats (HRs) 1 and 2 of SARS-CoV-2, has been relatively less studied despite its critical role in determining infectivity (Cai et al. 2020; Xia et al. 2020), and its distinctive features, evolutionary origin, and dynamics are still scarcely understood.

As for other class I fusion proteins (FPs), such as those found in other CoVs as well as in Orthomyxoviridae, Retroviridae, Paramyxoviridae, Filoviridae, and Arenaviridae (White et al. 2008), the fusion of SARS-CoV-2 with target cells depends on a complex structural transition of the trimeric spike protein by which, upon receptor binding and proteolytic cleavage, the exposed fusion peptide (FP) inserts in the cell membrane and a coiled coil (CC) 6-helix bundle (6-HB) is formed by tracts of HRs 1 and 2 (Rey and Lok 2018). This 6-HB FC, bringing together the host cell and viral lipid bilayers, is formed by a parallel CC trimer of HR1-derived helices (FC1) and by HR2-derived helices (FC2) of the three protomers, folding back onto the FC1 trimer and lying in the grooves between the FC1 helices, with antiparallel orientation (Cai et al. 2020). Early analyses of the SARS-CoV-2 spike protein sequence (e.g. Xia et al. 2020) revealed numerous clustered differences in its FC in comparison with that of SARS-CoV-1, i.e. the phylogenetically closest human CoV, and these differences were suggested to modify the H-bonding pattern. However, the overall structural and functional meaning of these highly clustered SARS-CoV-2 FC substitutions, also in relation to FCs of other CoV and non-CoV class I FPs, remains largely obscure.

The viral FC is subject to considerable selective pressure (e.g. Forni et al. 2015) given its fundamental role in driving the entry of the viral genome into target cells. Moreover, it can also contribute to antigenicity and to immune system evasion upon post-translational modification (Fung and Liu 2018; Cai et al. 2020; Grifoni et al. 2020; Shanmugaraj et al. 2020; Walls et al. 2020). Therefore, it is likely that evolutionary amino acid substitutions that accumulate in the FC may be critically linked to changes in the transition between the pre- and post-fusion spike conformations, in the stability of the post-fusion 6-HB, ultimately varying the transmission rate of the virus and its antigenicity. Analyses in the initial months of the human Covid-19 pandemic revealed several amino acid substitutions in the FC region with possible structural impact (e.g. Oliva et al. 2021). However, the evolutionary origin of the SARS-CoV-2 FC within Sarbecovirus CoVs, its phylogenetic history, current mutational trends, and their relationship, are poorly understood and still need to be traced.

To address these issues, we undertook a structure-guided investigation of the possible sequence/structure logic of the substitutions clustered within the FC that distinguish SARS-CoV-2 from the phylogenetically closest human CoVs, like SARS-CoV-1 and MERS-CoV. We found that these substitutions increase local HR stereotypy and dramatically shift the FC amino acid composition, leading to a considerable increase of the S/Q ratio, as observed in other highly syncytiogenic viruses, and to a striking alternate layering of S- and Q-rich regions along the post-fusion structure. Moreover, we found that the SARS-CoV-2 FC was already present in multiple Sarbecovirus species at least 15 years ago and likely appeared >50 years ago, spreading in both the SARS-CoV-2-like and SARS-CoV-1-like Sarbecovirus lineages likely by recombination. These findings shed new light on the remote origin of the SARS-CoV-2 fusion machinery and help rationalize in structural terms its mutational trends in the ongoing Covid-19 pandemic.

## 2. Results

### 2.1 Clustered amino acid substitutions in the SARS-CoV-2 FC1 occur in a spike protein region displaying low evolutionary variation and signatures of positive selection

**Figure 1.**
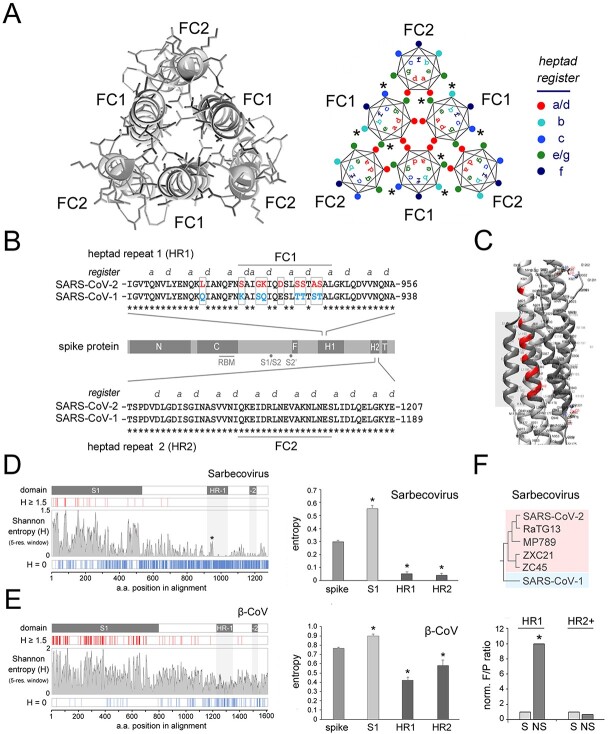
Evolutionary sequence variation in FC1/HR1 and signatures of positive selection. (A) *Left panel.* Detail of the post-fusion structure of the SARS-CoV-2 spike protein (PDB: 6XRA) encompassing the 6-HB formed by the antiparallel FC1 and FC2 helices of the three protomers, seen from a zenithal perspective. *Right panel.* Scheme of a CC 6-HB as in the left panel highlighting the relative positions of the residues in the seven positions of the heptad register (*a-b-c-d-e-f-g*) in each one of the six helices. (B) Schematic representation of the primary sequence of the SARS-CoV-2 spike protein (*light gray bar*) with the main functional domains highlighted in *dark gray* (*N* and *C*: N- and C-terminal portions of the S1 region, respectively; *RBM*: receptor binding motif; *S1/S2* and *S2ʹ*: protease cleavage sites at the junction between S1 and S2 and within S2, respectively; *F*: fusion peptide; *HR1*: heptad repeat 1; *HR2*: heptad repeat 2; *T*: transmembrane region). The alignments of the primary sequences of the SARS-CoV-1 and -2 spike proteins encompassing the FC formed by a portion of HR1 (FC1) and a portion of HR2 (FC2) from each protomer, are reported *above* and *below* the protein bar, respectively. The heptad register (positions *a* and *d* only) is highlighted above the alignments. The nine residues that differ between the HR1/FC1 sequences of SARS-CoV-1 and -2 are highlighted in *red* and *cyan*, respectively. (C) Detail of the SARS-CoV-2 spike protein post-fusion structure (PDB: 6LXT) encompassing the fusion core, from a lateral perspective. The nine residues that distinguish the SARS-CoV-2 FC (the *gray box* highlights a portion of it) from the SARS-CoV-1 FC are highlighted in *red*. (D) *Left panel*. The *central larger plot* displays the Shannon entropy value for each position in an amino acid sequence alignment of the spike proteins of 20 Sarbecoviruses, as listed in Zhang, Wu, and Zhang (2020), using a five-residue window (see *Methods*). The *thin vertical bars* above and below the entropy plot highlight those positions in the alignment with entropy ≥ 1.5 (*red*) or equal to 0 (*blue*). The *white bar* on top represents the spike protein primary sequence with domains of interest highlighted in *dark gray*. A *light gray* shading highlights the position of HR1 and HR2 along the entropy plot. Note how the clustered FC1 mutations are associated with a small peak in the entropy plot (*asterisk*). (E) As in *D* for an alignment of β-CoV spike proteins as reported in the phylogenetic tree by Lu et al. (2020) (see Fig. 7). A one-way ANOVA revealed overall significant differences between the mean entropy across the whole spike alignment (‘spike’) and some of its functional domains, such as the S1 region and the HRs 1 and 2 (*F*_(3,2569)_ = 34.37, *p *< 0.001). The same analysis showed that both HR1 and HR2 regions have a significantly lower entropy in comparison with the whole spike protein (*p* < 0.01, NK *post hoc* test in both instances), whereas the S1 region displays a significantly higher mean entropy (*p* < 0.05).

The SARS-CoV-2 FC is a 6-helix bundle (6-HB) formed by three central HR1-derived helices (FC1) and by three HR2-derived helices (FC2) with antiparallel orientation. Figure 1A (*left panel*) shows a detail of the post-fusion structure (Cai et al. 2020), from a zenithal perspective, and a scheme of the six coiled helices highlighting the heptad positions (*a, b, c, d, e, f, g*; Parry, Fraser, and Squire 2008) in a typical antiparallel CC hexamer (*right panel*).

At the primary sequence level, the HR1 of SARS-CoV-2 differs from that of SARS- CoV-1, the phylogenetically closest human CoV, only by nine amino acid substitutions, eight of which are strikingly clustered within the FC1 tract, and one is immediately upstream (Fig. 1B,C; Xia et al. 2020). Conversely, the HR2/FC2 sequences are identical in the two viruses (Fig. 1B).

To determine whether these highly clustered amino acid substitutions that distinguish the HR1/FC1 of SARS-CoV-1 and -2 may be simply related to neutral sequence divergence, or may have instead a possible functional meaning, thus arising from selective processes, we first analyzed the overall natural sequence variation along the spike protein within Sarbecovirus, the CoV parent clade of SARS-CoV-1/-2, and then searched for specific mutational signatures of selection in the HR1 region (Fig. 1D–F).

To quantify the overall sequence variation, we performed a Shannon entropy analysis (Strait and Dewey 1996) in alignments of Sarbecovirus spike protein sequences, which showed that HR1 and 2 are among the spike regions with the lowest mean entropy, consistent with the presence of functional constraints to their variation, as opposed, for instance, to the highly variable S1 domain (Fig. 1D, *left panel*). Indeed, a one-way ANOVA (Fig. 1D, *right panel*) revealed overall significant differences between the mean entropy across the whole spike alignment (‘spike’) and some of its functional domains, such as the S1 region and the HRs 1 and 2 (*F*_(3,1972)_ = 63.04, *p *< 0.001). The same analysis showed that both the HR1 and HR2 regions have a significantly lower entropy in comparison with the whole spike protein (*p* < 0.001, Newman-Keuls (NK) *post hoc* test, in both instances), whereas the S1 region displayed a significantly higher mean entropy (*p* < 0.001). Similar results were obtained when we extended our analyses also to α-/β-CoVs (Fig. 1E, Supplementary Fig. S1A) although, while the relative mean entropy (i.e. variability) of HR1 is higher than that of HR2 across Sarbecovirus, where HR2 is quite invariant, the mean HR2 entropy is higher than that of HR1 across β-CoVs and α/β-CoVs. This observation indicates that the relative variation rates of HR1 and HR2 are not constant within CoVs but rather reflect the evolutionary trajectories of the fusion machinery in the different viral clades.

Thus, the clustered FC1 substitutions in SARS-CoV-2 occur within a context of very low sequence variation in HR1, which suggests that they may be the result of selective pressure rather than the mere reflection of the HR1 neutral mutational rate across Sarbecovirus (Fig. 1F). To test this hypothesis, we performed a McDonald–Kreitman (MK) test (McDonald and Kreitman 1991), which detects signatures of selective pressure exceeding neutral sequence variation in gene regions. Before performing this analysis, we preliminarily ruled out substitution saturation according to the Xia and Lemey (2009) method (*Iss* = 0.41 *vs. Iss.c* 0.83, *p* < 0.0001; see *Methods*). By comparing the intra-clade and inter-clade sequence variation (e.g. Xu et al. 2018) in SARS-CoV-2-like viruses *versus* SARS-CoV-1 (Fig. 1F, *upper panel*), the MK test showed that the observed substitutions within HR1 exceed neutral expectations, indicating positive selection, as shown by a neutrality index (NI) well below 1 (NI = 0.10), with significant Fisher’s exact test (*p* < 0.01) and G test (*G* = 10.28, *p* < 0.01). Conversely, the MK test revealed a trend, although non-significant, toward purifying selection in alignments of comparable length of the C-terminal spike region (*HR2+*, see Methods) comprising HR2.

Taken together, the entropy analysis and the MK test concurrently support the conclusion that the observed amino acid substitutions in the HR1 region, that distinguish SARS-CoV-2 from SARS-CoV-1, may be function-related rather than simply the result of neutral sequence variation.

### 2.2 Clustered amino acid substitutions in the SARS-CoV-2 FC enhance local heptad stereotypy

**Figure 2.**
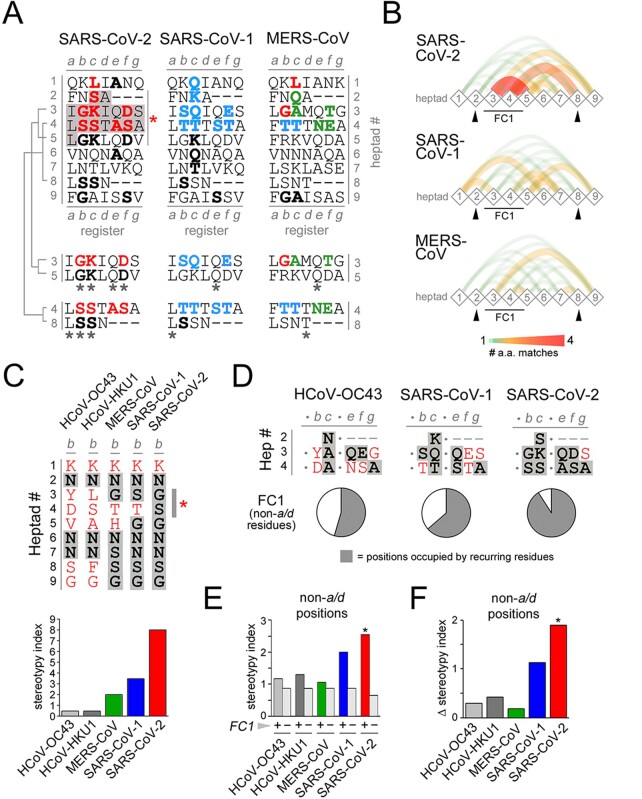
Heptad stereotypy in the FC1 and flanking heptads of the SARS-CoV-2 spike protein. (A) Primary sequences of the vertically aligned HR1 heptads flanking the FC1 region in SARS-CoV-2, SARS-CoV-1, and MERS-CoV (highlighted in *gray* for SARS-CoV-2), arbitrarily numbered from 1 to 9. Dashes correspond to shifts in the heptad register, i.e. *stutters* (Parry, Fraser, and Squire 2008). The residues highlighted in *red* and *cyan* for SARS-CoV-2 and SARS-CoV-1, respectively, correspond to the nine residues that distinguish the HR1/FC1 region of the two viruses, as in *panel B*. For MERS-CoV, the residues at the corresponding positions are highlighted in *green* when they are MERS-CoV-specific, whereas they are in *red* or *cyan* when they match those in the SARS-CoV-2 or SARS-CoV-1 FC, respectively. In all three sequences, residues belonging to heptads outside the FC1 that match *red, blue*, or *green* residues at the same heptad position are highlighted in *black*. The alignments of heptads 3/5 and 4/8 highlight the higher number of residue matches (*asterisks*) at the different heptad positions for SARS-CoV-2 in comparison with SARS-CoV-1 and MERS-CoV. (B) Graphs representing the pairwise heptad-to-heptad similarity scores, calculated as the sum of the residue matches at each heptad position. *Diamond-shaped nodes* represent the nine heptads and the thickness and color of the *curved edges* connecting them represent the magnitude of the similarity score according to the *legend* below. (C) *Upper panel*. Residues at position *b* in heptads 1–9 (as numbered in *panel A*) for the five human β-CoVs. For each virus (*columns*), residues appearing only once at position *b* in this heptad range are in *red*, whereas residues appearing multiple times are highlighted in *gray*. Note how most of the residues at position *b* in SARS-CoV-2 are occupied by recurring N, G, and S residues, whereas in HCoV-OC43 most of the residues, except for N, appear only once in this heptad range. *Lower panel*. Heptad stereotypy index at position *b* (heptads 1–9) for the viruses indicated in the *upper panel*. The increasing index from HCoV-OC43 to SARS-CoV-2 reflects the increasing proportion of recurring residues. (D) *Upper panels*. Residues at positions *b*/*c*/*e*/*f*/*g* (i.e. non-*a*/*d* positions) in the FC1 of the indicated viruses. For each position, residues appearing only at that position in the heptad range 1–9 (within or without the FC1 heptads) are in *red*, whereas residues appearing multiple times are highlighted in *gray*. Note how most of the residues at position *b*/*c*/*e*/*f*/*g* in SARS-CoV-2 are occupied by recurring residues, four of which are S. *Lower panel*. Pie charts displaying the overall relative proportion of recurring residues (*gray*) and residues occurring only once (*white*) at positions *b*/*c*/*e*/*f*/*g* in the FC1 of the indicated viruses, as shown in the *upper panel*. (E) Bar graph displaying the overall stereotypy index calculated at positions *b*/*c*/*e*/*f*/*g* for the indicated viruses, either taking into account (+) or not (−, *light gray*) the residues within FC1. (F) Bar graph displaying, for each indicated virus, the difference (Δ) between the overall stereotypy index at positions *b*/*c*/*e*/*f*/*g* calculated either taking into account or not the residues within FC1, as shown in *panel E*. Note how SARS-CoV-2 has the highest Δ value which reflects the considerable impact of FC1 residues in increasing local heptad stereotypy.

To better understand the possible structural and functional meaning of these clustered substitutions, we analyzed them in relation to their positions in the heptad register of HR1 (Fig. 2A) which, as for the SARS-CoV-1 spike (Supekar et al. 2004; Tripet et al. 2004), is characterized by two stutters around the FC1 section, by which the seven-residue periodicity is perturbed by four-residue inserts (Parry, Fraser, and Squire 2008). We found that, notably, seven of the nine residues that differentiate the SARS-CoV-2 from the SARS-CoV-1 FC1 are in heptad positions *b, c*, and *e* (Fig. 1A, *right panel*, asterisks) where they can potentially engage in inter-helical interactions with residues in *e, g*, and *a* positions of FC2.

Strikingly, the SARS-CoV-2-specific substitutions drive a considerable increase in the sequence similarity between the FC1 and the flanking heptads, in comparison with what can be observed in SARS-CoV-1 and in the more distantly related MERS-CoV (Fig. 2A). Thus, for instance, the SARS-CoV-2-specific residue doublets ‘GK’ and ‘SS’ at positions *b*/*c* in heptads 3 and 4 match identical doublets in heptads 5 and 8, respectively. Similarly, three SARS-CoV-2 specific FC1 residues aligned in position *c* in heptads 2, 3, 4 form, with the K in heptad 5, a vertical S-K-S-K motif, and the D/S residues at position *f* in heptads 3/4, respectively, match D or S residues at the same position in heptads 5 and 9. Alanine in position *e* in heptad 4 within FC1 matches two alanine residues at the same position in heptads 1 and 6 (see also Supplementary Fig. S1B which shows the frequent recurrence of alanine at *e*/*g* positions in human CoVs). Figure 2B highlights the amino acid identities between heptad pairs in SARS-CoV-2 *versus* those in SARS-CoV-1 and MERS-CoV. Similar results were obtained even when considering an uninterrupted heptad register (i.e. with no stutters), given the fact that the region of increased stereotypy is comprised between the two stutters upstream and downstream of the FC1 sequence (Supplementary Fig. S1C–D; see *Discussion*).

Overall, SARS-CoV-2 displays 15 pairs of matching amino acids in the same register positions in heptad pairs across heptads 1–9, which is 1.87 and 3.75 times more than what found in SARS-CoV-1 and MERS-CoV, respectively (Supplementary Fig. S1E). Moreover, the vast majority of these matches in SARS-CoV-2 FC1 come from heptads with higher pairwise similarity (i.e. two or more matches), indicating again higher degrees of heptad similarity in SARS-CoV-2 versus both SARS-CoV-1 and MERS-CoV (Supplementary Fig. S1F). As for the previous findings, also these differences were conserved when considering a continuous heptad register without taking into account stutters (Supplementary Fig. S1G–H).

Furthermore, we also developed a stereotypy analysis for each heptad position, which helped highlight inherent trends in the evolution of human CoVs (Fig. 2C–E). Thus, we calculated a ‘stereotypy index’ to measure the diversity in amino acid composition at each heptad position for each virus. We included in this analysis FC1 and flanking heptads (1–9), as in Fig. 2A, for the five human β-CoVs (as the two α-CoVs have larger, non-directly comparable FCs). Specifically, at a given position in the register (e.g. ‘*b*’; Fig. 2C) in the nine analyzed heptads, we counted for each virus (1) the number of heptads in which the given position was occupied by amino acids recurring more than once across the nine heptads (*r*) and (2) the number of heptads occupied instead by amino acid residues occurring only once (*o*). The stereotypy index was calculated as the *r*/*o* ratio at each position for each virus. Thus, Fig. 2C highlights how the stereotypy index at heptad position *b* (*upper panel*) progressively increases from HCoV-OC43 to SARS-CoV-2 (*lower panel*). Notably, both amino acids at position *b* in the SARS-CoV-2 FC (i.e. G and S; *upper panel, asterisk*) also recur repeatedly at the same position in the flanking HR1 heptads. This was also the case for most of the other non-*a*/*d* heptad positions (Fig. 2D). *A*/*d* positions were not included in this analysis as they are by definition stereotypical in CC sequences, containing mostly hydrophobic residues, typically leucine in ‘leucine zippers’. Indeed, ten of the eleven amino acids, i.e. ∼90 per cent, at *b*/*c*/*e*/*f*/*g* positions within the SARS-CoV-2 FC recurred, at the corresponding positions, in the flanking heptads (Fig. 2D*, right panel and pie chart*). Conversely, this percentage drops to ∼63 per cent and ∼54 per cent in the SARS-CoV-1 and HCoV-OC43 FC1, respectively (Fig. 2D*, left and middle panels and pie charts*). Overall, when calculating the stereotypy index by pooling all the non-*a*/*d* heptad positions (i.e. *b*/*c*/*e*/*f*/*g*), we found that SARS-CoV-2 had the highest index among the five human β-CoVs (Fig. 2E, *colored bars*), paralleling what observed for position *b* alone (Fig. 2C). Remarkably, the stereotypy index of SARS-CoV-2 was instead lower than that of the other viruses when it was calculated without including the FC1 heptads (Fig. 2E, *colored bars*). Indeed, SARS-CoV-2 displayed the highest difference (Δ) in stereotypy index when calculated with or without the contribution of the FC1 heptads (Fig. 2F), which was statistically significant (*p* < 0.02, *χ*^2^ test). Such significant difference was not observed for the other human β-CoVs (*p* > 0.13 in all instances).

Taken together, these results indicate that the nine amino acid substitutions that distinguish the SARS-CoV-2 from the SARS-CoV-1 FC1 sequence drive a considerable increase in the local heptad stereotypy, which in SARS-CoV-2 reaches the highest level among the human β-CoVs.

### 2.3 Serine enrichment in the FC and alternate S/Q layering in the SARS-CoV-2 spike post-fusion structure

**Figure 3.**
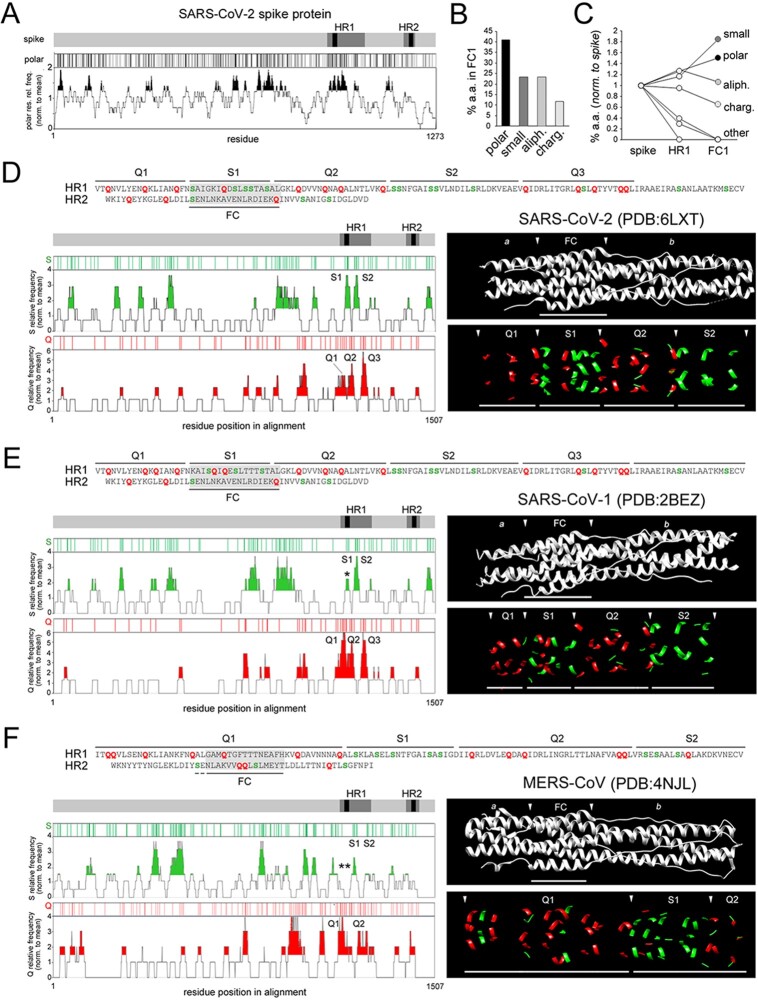
A positional, compositional, and structural analysis reveals a S-rich FC and an alternate S/Q layering along the SARS-CoV-2 spike protein post-fusion structure. (A) The *gray bar* represents the primary sequence of the spike protein with the HR1/2 regions, and the FC1/2 segments within them, highlighted in *dark gray* and *black*, respectively. Below, the *black* vertical bars mark the position of individual polar residues (N, Q,S, T), and the graph plots the relative frequency of polar residues along the spike protein sequence (calculated using a 21-residue sliding window), normalized to the overall occurrence of polar residues in the spike primary sequence. Peaks of local polar residue overrepresentation above 1.25 times the protein-wide occurrence of polar residues are highlighted in *black*. (B) Bar graph plotting the percent occurrence in FC1 of amino acids belonging to different classes, i.e. *polar* (N, Q, S, T), *small* (A, G), *aliphatic* (I, L, V) and *charged* (D, E, H, K, R). (C) Graph plotting the percent occurrence of amino acids belonging to the indicated classes either in the whole spike protein, or in HR1, or in FC1. Values are normalized to those in the whole spike protein. The amino acid classes *polar, small, aliphatic*, and *charged* are as in *panel B*. Other classes, i.e. aromatic (F, W,Y), sulphurated (C, M) and cyclic (P), which are absent in FC1, are collectively indicated as *other.* (D) *On top*, part of the SARS-CoV-2 HR1 sequence and the reverse sequence of HR2. The two sequences are horizontally aligned so to reproduce approximately the antiparallel overlap of the FC1 and FC2 segments in the 6-HB (highlighted in *gray*). Q and S residues are highlighted in *red* and *green*, respectively. Q- and S-rich portions of the HRs/FC are marked by *black segments* and numbered progressively. The *gray bar* represents the primary sequence of the spike protein with the HR1/2 regions, and the FC1/2 segments within them, highlighted in *dark gray* and *black*, respectively. Below the bar are reported plots representing the position of individual S and Q residues (*green* and *red vertical bars*, respectively), and graphs plotting the relative frequency of S and Q residues along the spike protein sequence, when aligned with that of the SARS-CoV-1 and MERS-CoV spike proteins. Values in these graphs are normalized to the overall occurrence of S or Q residues, respectively, in the spike primary sequence. Peaks of S or Q local overrepresentation above 1.5 times the protein-wide S or Q occurrence are highlighted in *green* or *red*, respectively. The peaks identifying S- and Q-rich regions of interest in the HR1/2 regions (>2 for the S graph, and >3 for the Q graph) are numbered progressively (S1, S2,… and Q1, Q2,…) and correspond to those marked on the primary sequence above. The ribbon structure images *on the right side* show the overall post-fusion structure *in black and white* (*upper panel*) with the FC region delimited by *arrowheads* (*a* and *b* label the regions upstream and downstream of the FC, respectively). The *lower panel* shows the same structure but only with Q (*red*) and S (*green*) residues visible. *Arrowheads* delimitate the indicated Q- and S-rich regions. (E-F). As in *panel A* for the SARS-CoV-1 and MERS-CoV spike proteins, respectively.

Another striking aspect of the nine substitutions that differentiate the SARS-CoV-2 from the SARS-CoV-1 HR1/FC1 is the fact that in five cases they involve serine (S) residues, leading to a dramatic increase in the percentage of S residues in the SARS-CoV-2 FC1, which reaches ∼30 per cent. As a consequence, polar residues (N/Q/S/T) represent >40 per cent of the FC1 residues and thus they are the quantitatively more conspicuous amino acid class in this region (Fig. 3A,B; the list of amino acids in each class in reported in the figure legend). Moreover, polar residues are also among those more overrepresented in the FC1 in comparison with the whole HR1 or the entire spike, together with small residues (whose higher occurrence is related to alanine residues at positions *e*/*g*; Fig. 3C; Supplementary Fig. S1B). Other groups are either not overrepresented in FC1 (aliphatic) or underrepresented (charged). Remarkably, other classes are almost completely excluded (aromatic, sulphurated) or absent (cyclic) from HR1/FC1. For these reasons, we focused our analysis on the possible consequences of the FC1 enrichment in serine and polar residues that are overrepresented in both absolute and relative terms in this region of the spike protein.

These observations prompted us to investigate whether the observed serine enrichment is a specific feature of the FC1 region or simply a reflection of a generalized compositional bias in the entire spike protein of SARS-CoV-2. Thus, we undertook a systematic analysis of the distribution of serine and other residues along the SARS-CoV-2 spike sequence, quantifying the local enrichment of the amino acids using a 21-residue sliding window, and normalizing the local percent occurrence of each amino acid to its overall percent occurrence in the entire spike protein (Fig. 3D–F). This analysis revealed that, within the SARS-CoV-2 spike protein, FC1 corresponds to a local peak of high S frequency (S1), in which S residues occur almost four times more frequently than in the rest of the protein (Fig. 3D, *lower left panels*). A second, similar peak (S2) occurs downstream in the HR1 region. Strikingly, these peaks alternate with three distinct glutamine (Q) frequency peaks (Q1–Q3) within HR1. This alternation of S- and Q-rich regions within the HR1 primary sequence is clearly detectable along the post-fusion spike protein structure (PDB: 6LXT; Fig. 3D, *lower right panels*). Remarkably, S and Q residues from the antiparallel HR2/FC2 region also contribute to the overall S/Q layering along the longitudinal axis of the post-fusion structure (Fig. 3D, *upper panel*).

A comparative analysis of the SARS-CoV-1 and MERS-CoV spikes showed that the S1 peak associated with FC1 is either considerably blunted in SARS-CoV-1 (Fig. 3E, *lower left panels asterisk*) or absent in MERS-CoV (Fig. 3E, *lower left panels, double asterisk*). Thus, while in the SARS-CoV-1 post-fusion spike structure the FC1 region displays a combined S/Q enrichment (PDB: 2BEZ; Fig. 3E, *lower right panels*), in the corresponding MERS-CoV structure a broadened Q enrichment peak (Q1) comprises the FC1 region, where only a single S residue is present (PDB: 4NJL; Fig. 3F, *lower right panels*). Also for the SARS-CoV-1 and MERS-CoV viruses, the overall S/Q layering in the post-fusion spike structure depends on S/Q residues in the antiparallel HR2/FC2 (Fig. 3E–F, *upper panels*).

These findings show that the clustered amino acid substitutions in HR1/FC1 critically alter the distribution of polar amino acids in the SARS-CoV-2 FC, leading to a high enrichment in serine, and contribute to the establishment of a sharp, alternate S/Q layering along the post-fusion structure which can be found, although variably blunted or modified, in SARS-CoV-1 and MERS-CoV.

### 2.4 S/Q and (S + N)/(Q + T) ratios define three types of FCs in class I FPs

The previous findings prompted us to determine whether the enrichment in serine and the alternate layering of S- and Q-rich spike regions are exclusive features of SARS-CoV-2 or whether they can be detected in the available structures of other class I FPs. Thus, we analyzed the available post-fusion structures of spike proteins of other hCoVs and several representative viruses infecting humans, including emerging pathogens with zoonotic origin. These latter viruses are representative of the main viral families bearing class I FPs (White et al. 2008), i.e. Orthomyxoviridae (i.e. influenza virus), Paramyxoviridae (i.e. respiratory syncytial virus (RSV), Newcastle Disease virus (NDV), Nipah virus (NiV), Hendra virus (HeV), mumps virus (MuV)), Retroviridae (i.e. HIV), Filoviridae (i.e. Ebola virus, (EBOV)), and Arenaviridae (i.e. Lassa virus, (LASV)).

HCoV-NL63 and HCoV-229E are the only human CoVs, besides SARS-CoV-2, SARS-CoV-1, and MERS-CoV, whose post-fusion crystal structures are available (Supplementary Fig. S2). These two viruses, which belong to α-CoVs, have a larger FC than SARS-CoV-2 and other β-CoVs. In compositional terms, their FC displays intermediate features between those of SARS-CoV-2 (S-rich) and MERS-CoV (Q-rich), with less neat patterns of post-fusion S/Q layering, similar to what found in the SARS-CoV-1 post-fusion structure.

Interestingly, a sharper segregation of S and Q residues, similar to the SARS-CoV-2 one, can also be observed in the post-fusion structures of the FPs of other non-CoV human viruses (Supplementary Fig. S3). Thus, for instance, the post-fusion conformation of the NDV F protein shows at one end a S-rich 6-HB FC followed by a central Q-rich CC trimer, and, at the other end, a second S-rich region that encompasses even non-helical regions.

**Figure 4.**
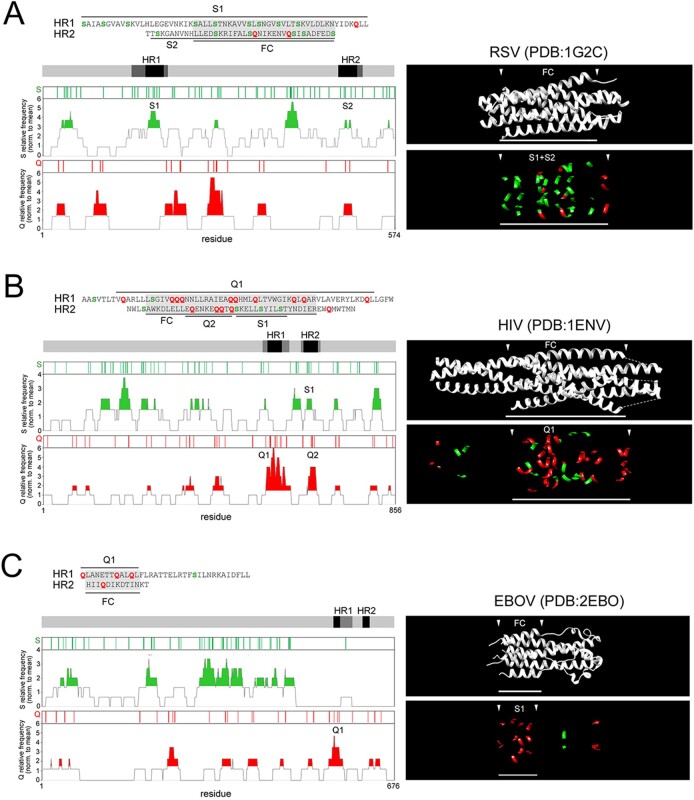
S-rich and Q-rich FCs in class I FPs. A-C. As in Fig. 2D for RSV, HIV, and EBOV. Here, the horizontal axis in the Q and S frequency plots corresponds to the actual residue positions in the non-aligned primary protein sequences that are indicated.

The analysis of other class I FPs revealed overall a compositional gradient of their FCs, ranging from S-rich ones, like in RSV, NDV, and SARS-CoV-2, to Q-rich ones, like in HIV, LASV, and EBOV, with intermediate grades (Fig. 4; Supplementary Fig. S3).

**Figure 5.**
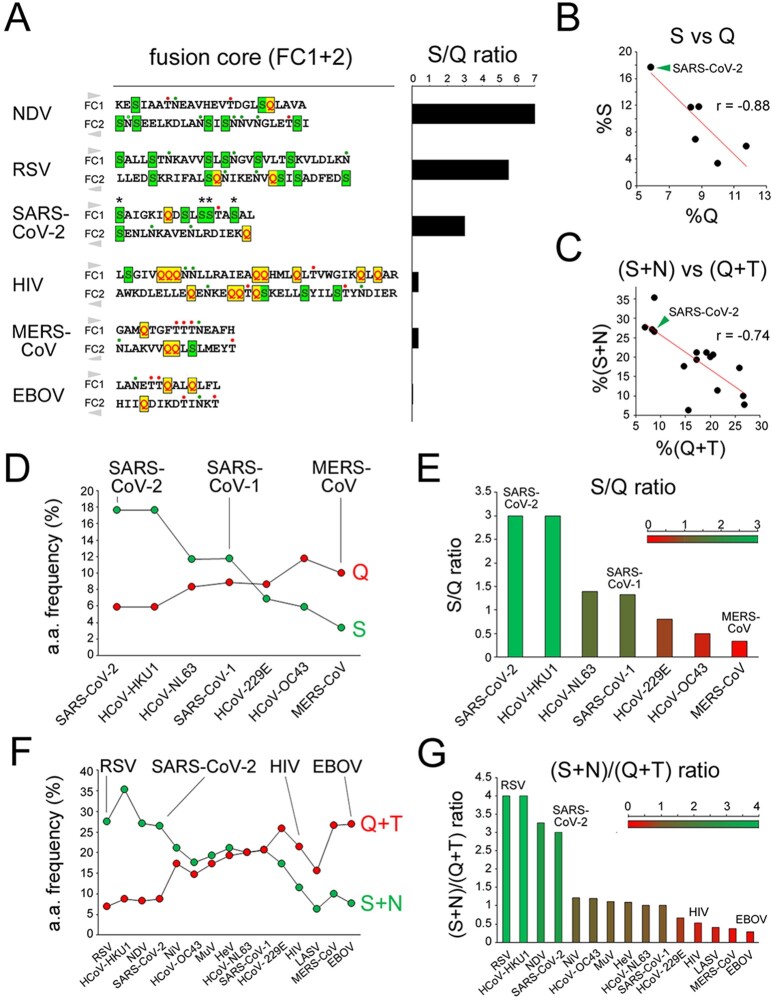
The S/Q and (S + N)/(Q + T) ratios categorize CoV and other class I FPs. (A) *Left*. Primary sequences of the juxtaposed FC1 and FC2 segments for the indicated viruses. The FC2 sequences are reversed to reproduce the antiparallel arrangement of the FC1 and FC2 helices in the post-fusion structure. S residues are highlighted in *dark green*, within *light green* boxes, Q residues in *red*, within *yellow* boxes. N and T residues are marked by *green* and *red* dots, respectively, above the sequences. Note how four S residues (*asterisks*) in the SARS-CoV-2 FC are among the nine residues that differentiate it from the SARS-CoV-1 FC. *Right*. Bar graph plotting the S/Q ratio in the same FCs as shown on the *left*. (B) Scatterplots representing the correlation between the percent occurrence of S and Q residues in the FCs of the seven human CoVs. The *green arrowhead* indicates the SARS-CoV-2 datapoint. (C) Scatterplots representing the correlation between the percent occurrence of S + N and Q + T residues in the FCs of the seven human CoVs and other representative viruses with class I FPs. The *green arrowhead* indicates the SARS-CoV-2 datapoint. (D) Graph plotting the percent occurrence of S and Q residues in the FCs of the indicated human CoVs. (E) Bar graph plotting the S/Q ratio in the FCs of the indicated human CoVs. (F) Graph plotting the percent occurrence of S + N and Q + T residues in the FCs of the indicated viruses with class I FPs. (G) Bar graph plotting the (S + N)/(Q + T) ratio in the FCs of the indicated viruses with class I FPs.

We quantified the ratio between the percentages of S and Q in the combined FC1 and FC2 primary sequences (Fig. 5A; Supplementary Fig. S4A) and we found that these two percentages have a significant inverse correlation across hCoVs (*r* = −0.88, *n* = 7, *p* < 0.01; Fig. 5B). Interestingly, the occurrences of the other two polar residues (asparagine, N, and threonine, T) were differentially linked to those of S and Q. Indeed, across hCoV and other class I FCs, the N occurrence negatively correlates with the Q occurrence (*r* = 0.55, *n* = 15, *p* < 0.03; Supplementary Fig. S4B), and the T occurrence also displayed a trend, although non-significant, toward a negative correlation with the S occurrence (Supplementary Fig. S4C). Thus, overall, the %(S + N) and %(Q + T) in the FC were also inversely related in hCoVs and other class I FPs (*r* = −0.74, *n* = 15, *p* < 0.01; Fig. 5C). Therefore, based on their S/Q or (S + N)/(Q + T) ratios (Fig. 5D–G, respectively), the FCs in class I FPs can be classified into S-rich (ratio ≥ 2; e.g. NDV, RSV, SARS-CoV-2, HCoV-HKU1), Q-rich (ratio ≤ 0.5; e.g. HIV, MERS-CoV, LASV, EBOV), or intermediate (ratio between 0.5 and 2).

These results indicate that the clustered substitutions in the SARS-CoV-2 FC, leading to the net gain of four S residues and loss of one Q with respect to the SARS-CoV-1 FC, induce a considerable change in the local amino acid composition towards a highly S-rich configuration. Remarkably, such FC composition is similar to that of highly syncytiogenic viruses with respiratory tropism, such as RSV and NDV, which is of considerable interest given the high propensity of SARS-CoV-2 to induce the formation of syncytia in infected lungs (Musarrat et al. 2020; Ou et al. 2020; see *Discussion*).

### 2.5 Impact of polar residue enrichment in the FC on post-fusion H-bonding patterns

**Figure 6.**
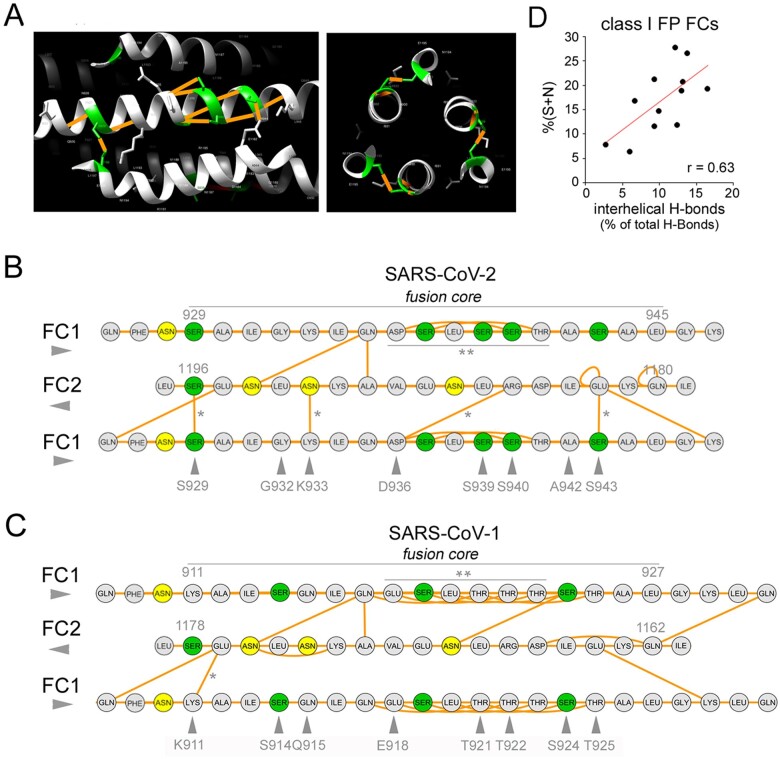
Intra- and inter-helical H-bonds in the FCs of SARS-CoV-2 and other viruses with class I FPs. (A) *Left*. Detail (lateral view) of the SARS-CoV-2 spike post-fusion structure (PDB: 6LXT) showing a portion of the HR1/FC1 (*central* helix) and two portions of the flanking FC2 helices (*upper* and *lower* helices). S residues are highlighted in *green*, and H-bonds involving these residues in *orange. Right*. Detail (zenithal view) of the same structure highlighting the ‘polar layer’ formed by S929 and S1196, establishing S-to-S H-bonds in the three protomers. (B) Graph representation of the H-bond network in the SARS-CoV-2 post-fusion FC (PDB: 6LXT). For simplicity, only one FC2 tract (residues 1180–1196) and two flanking, antiparallel FC1 tracts (residues: 929–945) are depicted, with a few flanking residues. S and N residues are highlighted in *green* and *yellow*, respectively. *Orange* segments represent H-bonds. *Single asterisks* represent inter-helical H-bonds formed by residues of the SARS-CoV-2 FC that are not present in the SARS-CoV-1 FC (indicated by *gray arrowheads*). The *double asterisk* signals a S-rich stretch with a relatively dense network of intra-helical H-bonds. (C) As in *panel B*, for the SARS-CoV-1 FC. *Gray arrowheads* signal residues that differ from SARS-CoV-2. Note how the T-rich stretch (*double asterisk)* forms an even denser network of intra-helical H-bonds than the corresponding S-rich stretch in SARS-CoV-2. (D) Scatterplot displaying the positive correlation between the percent occurrence of S + N residues and the percentage of interhelical H-bonds on the total number of H-bonds in the FCs of human CoVs and other viruses with class I FPs.

As polar residues are often involved in the formation of CC-stabilizing H-bonds, we analyzed whether the clustered FC substitutions that distinguish SARS-CoV-2 from SARS-CoV-1 have any impact on H-bonding patterns, and whether, more in general, S/N-rich and Q/T-rich FCs differ with respect to H-bonding. When we analyzed distance-based H-bond predictions in the post-fusion structure of SARS-CoV-2 (PDB: 6LXT), we found that amino acids at four of the eight residues that distinguish the FC1 of this virus from the SARS-CoV-1 one, are involved in inter-helical H-bonds between FC1 and FC2 helices (Fig. 6A–B). Importantly, two of these are serine residues. Thus, S929 in FC1 establishes a H-bond with S1196 in FC2, and the three S929-S1196 H-bonds that are formed in the structure resemble a ‘polar layer’ (Duquerroy et al. 2005; Suntoke and Chan 2005; Fig. 6A, *right panel*) stabilizing the CC hexamer. Moreover, S943 forms an H-bond with E1182. The other two inter-helical bonds involving these residues are K933-N1192 and D936-R1185. The other two S residues in the SARS-CoV-2 FC1 that are not present in the SARS-CoV-1 FC are contiguous (S939-S940) and both establish side-chain-to-backbone H-bonds that can stabilize the FC1 helix. We found that, interestingly, similar S doublets recur not only along the SARS-CoV-2 HR1 sequence (S967-S968; S974-S975) but also in the HRs/FCs of other viruses such as HeV and NiV (Supplementary Fig. S4A). Side-chain-to-backbone H-bonds may be a defining feature of CC structures rich in polar residues (Escobedo et al. 2019) and may critically contribute to their stability. It is also noteworthy that two out of three asparagine residues in the FC are involved in interhelical H-bonds (i.e. N1194 with Q935, and N1192 with K933).

In comparison with SARS-CoV-1 (Fig. 6C), the eight substitutions within the FC that characterize SARS-CoV-2 lead to a net increase of three inter-helical H-bonds in the post-fusion hexamer. Indeed, two substitutions K933 and S943 in SARS-CoV-2 for Q915 and T925 in SARS-CoV-1, respectively, introduce six more inter-helical H-bonds in the 6-HB structure, whereas the substitution of S924 in SARS-CoV-1 by alanine (A942) in SARS-CoV-2 leads to a loss of three inter-helical bonds. Other two substitutions simply replace existing inter-helical bonds (S929-S1196 and D936-R1185 in SARS-CoV-2 replace K911-E1176 and E918-R1167 in SARS-CoV-1, respectively), and the other three (S914, T921, and T922 in SARS-CoV-1 with G932, S939, and S940 in SARS-CoV-2, respectively) not lead to any loss or gain of inter-helical bonds.

While the overall change in the total number of H-bonds was not conspicuous, it should be considered that in viral FCs the substitution of even a single residue altering just one single inter-helical interaction can disrupt both FC stability and the infectivity of viral particles (e.g. He et al. 2007); Lu et al. 1999. Notably, the clustered substitutions considerably altered the H-bond distribution pattern within FC1. Indeed, four FC1 substitutions in SARS-CoV-2 (i.e. S929, K933, D936, S943) insert four quite evenly spaced H-bonds along the FC1/FC2 interface (Fig. 6B, *asterisks*), whereas SARS-CoV-1 has only one (K911) at the corresponding positions. Moreover, five substitutions that differentiate the SARS-CoV-2 FC1 region 936–943 from the corresponding SARS-CoV-1 region (918–925) not only introduce two inter-helical H-bonds (D936, S943) but also reduce the number of intra-helical side-chain-to-backbone H-bonds, mostly established by threonine residues (T921, T922, T923, T925), three of which are replaced by serine/alanine in SARS-CoV-2.

The fact that five out of nine S/N residues within the SARS-CoV-2 FC are involved in interhelical H-bonds prompted us to explore whether the relative enrichment in these two residues in viral FCs may correlate with the density of inter- *versus* intra-helical H-bonds. Thus, we calculated (1) the total percentage of S + N residues and (2) the percentage of interhelical over the total H-bonds, and their correlation, in 13 viruses bearing class I FPs with available post-fusion structures (Fig. 6D). This analysis revealed a significant correlation between the S/N content and the proportion of interhelical H-bonds in class I FCs (*r* = 0.63, *n* = 13; *p* < 0.03).

Taken together, these findings show that the substitutions leading to the increased S/Q ratio in the SARS-CoV-2 FC directly impact H-bonding patterns, and therefore the stability of the 6-HB. Furthermore, they reveal a more general structural correlate of the S/Q ratio in class I FCs, indicating that increasing proportions of S and N residues may favor the formation of inter-helical over intra-helical H-bonds, which can have a considerable structural impact.

### 2.6 The SARS-CoV-2 FC is present in multiple SARS-CoV-2-like and SARS-CoV-1-like Sarbecoviruses

**Figure 7.**
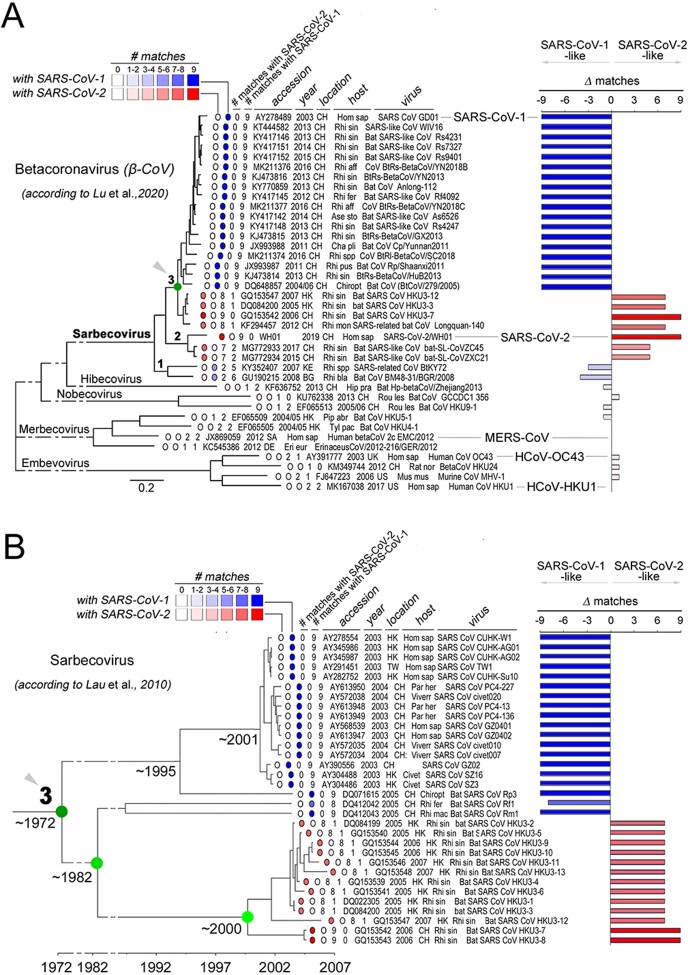
SARS-CoV-2-like and SARS-CoV-1-like FCs in β-CoVs and in the Sarbecovirus subgenus. (A) Phylogenetic tree of β-CoV full-length genomes, as derived from Lu et al. (2020) with minor simplifications. For each one of the indicated viruses, the NCBI sequence ID and related information (legend *on top*), are reported, together with two numerical scores (from 0 to 9) representing the count of the residue matches in FC1 sequence alignments with either SARS-CoV-2 or SARS-CoV-1, at the nine positions that differentiate the FC1 region of these two latter viruses (see Fig. 1B). The host species name abbreviations (also for panel B) are reported in the Methods section. The numbers 1, 2, 3 indicate clades of the Sarbecovirus subgenus, as identified by Lu and coll. The *green node* (*3*) identifies the same clade as the one analyzed in *panel B*. The bar graph *on the right side* indicates the difference (Δ) between the two calculated scores, ranging from −9, as for viruses with a SARS-CoV-1 FC1, to 9, as for viruses with a SARS-CoV-2-like FC1. Viruses with either no similarity or modest, non-preferential similarity (e.g. MERS-CoV) at the nine positions with either SARS-CoV-1 or -2 tend to have a Δ ∼0. (B) As in A, for the Sarbecovirus phylogenetic tree derived from Lau et al. (2010), based on ORF1ab alignments, with minor simplifications. The *dark green node* (*3*) corresponds to the same node in *panel A*. The time bar reported below the tree is derived as well from Lau et al. (2010) and represents the estimated divergence times between the major clades reported in the tree based on a most recent common ancestor (MRCA) analysis. The main bifurcations in the evolutionary path that brought to viruses with a SARS-CoV-2-like FC are highlighted in *shades of green.*

**Figure 8.**
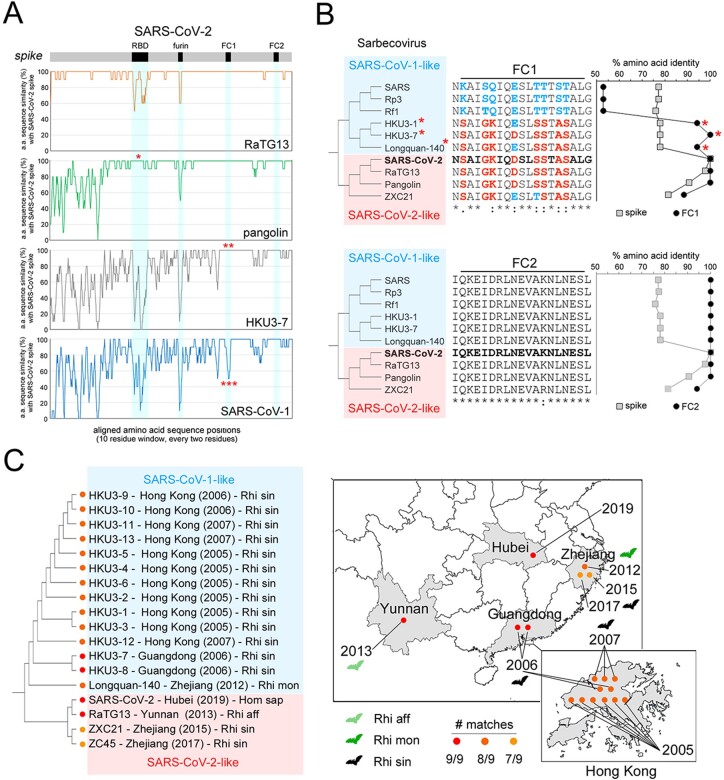
Phylogenenesis and phylogeography of the SARS-CoV-2 FC. (A) RAT sequence similarity plots of the aligned spike proteins of the indicated viruses in comparison with the SARS-CoV-2 spike, which is schematized as a *gray bar* above the graphs, with the *RBD, furin cleavage site* and *FC1/2* regions highlighted in *black*. The *single asterisk* highlights the similarity of the SARS-CoV-2 RBD with the pangolin CoV RBD. The *double asterisk* highlights the identity of HKU3-7 FC1 with the SARS-CoV-2 FC1. The *triple asterisk* highlights the difference between the SARS-CoV-1 FC1 and the SARS-CoV-2 FC1. (B) *Upper section. On the left*, unscaled phylogenetic tree of representative SARS-CoV-1-like (*cyan box*) and SARS-CoV-2-like (*pink box*) Sarbecoviruses. Note how some of the SARS-CoV-1-like viruses (*red asterisks*) have a SARS-CoV-2-like FC1 sequence (*middle alignment*). The graph *on the right side* represents the degree of similarity, expressed as % amino acid identity in sequence alignments, between the entire spike sequence (*gray squares*) of the viruses in the tree and the spike sequence of SARS-CoV-2, or between the FC1 (*black circles*) of the same viruses and the SARS-CoV-2 FC1. Note how for three SARS-CoV-1-like viruses (*red asterisks*) the degree of similarity of the FC1 sequence with SARS-CoV-2 largely exceeds the overall spike similarity, while the opposite is true for the other SARS-CoV-1-like viruses. *Lower section*. As in the upper section for the FC2 region. (C) Unscaled phylogenetic tree of viruses with a SARS-CoV-2-like FC with their site and date of isolation, and host species (Rhi aff: *Rhinolophus affinis*; Rhi mon: *Rhinolophus monoceros;* Rhi sin: *Rhinopholus sinicus*; Hom sap: *Homo sapiens*). The maps on the right locate the geographical regions where these viruses have been isolated. The location of the specimen collection sites within each region is purely indicative and is not geographically accurate. The Hong Kong map was obtained from *d-maps* (https://d-maps.com/carte.php?num_car=137949&lang=en). The China map was obtained from *Vemaps* (https://vemaps.com/china/cn-02).

Based on the previous findings, we asked whether the S-rich FC, with the clustered substitutions that differentiate SARS-CoV-2 from SARS-CoV-1, is also present in other non-human α- and β-CoVs. Thus, we quantified, in CoV spike sequence alignments, the number of amino acid matches between each virus and SARS-CoV-2 or SARS-CoV-1 at the nine HR1 positions that differentiate these two latter viruses. We included in this analysis representative CoVs, as reported in available phylogenetic trees of α-/β-CoVs (Zhang, Wu, and Zhang 2020; Supplementary Fig. S5), β-CoVs (Lu et al. 2020; Fig. 7A), and of the β-CoV Sarbecovirus subgenus, to which both SARS-CoV-1 and SARS-CoV-2 belong (Lau et al. 2010; Fig. 7B). For each non-human CoV, we also calculated the difference (Δ) between the number of matches at the nine positions with SARS-CoV-2 and SARS-CoV-1. This analysis revealed, as expectable, that the highest number of matches with SARS-CoV-1 and -2 can be found in viruses belonging to the Sarbecovirus subgenus of β-CoVs, whereas members of the Hibecovirus, Nobecovirus, Merbecovirus (including MERS-CoV), and Embecovirus subgenera displayed either none or just 1–2 of the 9 possible matches with SARS-CoV-2 or SARS-CoV-1 (Fig. 7A; Supplementary Fig. S5). Sarbecoviruses displayed an essentially dicotomic distribution of SARS-CoV-2-like and SARS-CoV-1-like FCs (Fig. 7A–B). Indeed, while members of the more basal Sarbecovirus clade-1, as defined by Lu et al. (2020), appear to be more SARS-CoV-1-like (5–6 matches with SARS-CoV-1, 2 with SARS-CoV-2), viruses belonging to clades 2–3 are either entirely SARS-CoV-1-like (9 matches) or essentially SARS-CoV-2-like (7–9 matches). Strikingly, the viruses bearing a SARS-CoV-2-like FC include not only those that were recognized early on as direct precursors of the SARS-CoV-2 virus (e.g. the bat RaTG-13 or the pangolin MP789 CoV), but also a number of other viruses in clade-2 (ZC45, ZXC21) and in clade-3 (Longquan-140, HKU3-7, and other 12 members of the HKU3 clade). The latter finding is particularly remarkable, as the clade-3 CoVs with a SARS-CoV-2-like FC bear spike proteins that are SARS-CoV-1-like in any other respect. Indeed, sequence similarity plots (Fig. 8A–B) comparing the RaTG-13, pangolin, HKU3-7, and SARS-CoV-1 spike proteins to the SARS-CoV-2 one, show that HKU3-7 bears an FC1 sequence which is identical to the SARS-CoV-2 one, but diverges in the rest of the spike protein, which is instead more similar to the SARS-CoV-1 spike. Figure 8A also highlights similarities and differences of the same viruses with SARS-CoV-2 for other three key determinants of viral tropism and infectivity, i.e. the receptor binding domain (RBD), the furin cleavage site and the FC2 sequence, which, remarkably, all display distribution patterns that are distinct from the FC1 one. Thus, while FC2 is identical in all four viruses, none of them bears a furin cleavage site and only the pangolin virus shares a highly similar RBD with SARS-CoV-2, as noted early on upon isolation of the virus (Wu et al. 2020; Zhang, Wu, and Zhang 2020).

Taken together, these findings indicate that the SARS-CoV-2 S-rich FC is not a unique feature of this virus, representing instead a structural module that is present, as such or with minor modifications, across a range of Sarbecoviruses that are not directly related in phylogenetic terms. These observations are highly consistent with the notion that the CoV spike gene is a modular structure whose parts can reshuffle by recombination between different viral genomes (Graham and Baric 2010) and prompted us to explore the possible evolutionary origin of the SARS-CoV-2 FC.

### 2.7 Evolutionary history of the SARS-CoV-2 FC reveals remote origin and likely recombination-driven spread

A phylogeographic analysis of the available spike protein sequences of viruses bearing a SARS-CoV-2 FC indicates that this viral fusion module has been circulating among multiple species of viruses infecting bats of the *Rhinolophus* genus for at least 14 years before the isolation of SARS-CoV-2 in 2019 (Fig. 8C, *left panel*). Indeed, already in 2005–2006, SARS-CoV-2-like FCs were found in viruses such as HKU3-7, -8, and several others (Lau et al. 2010) isolated from *Rhinolophus sinicus* in Hong Kong and in the adjacent Guangdong province of China (Fig. 8C, *right panel*). Subsequently, SARS-CoV-2-like FCs were repeatedly detected from 2012 to 2017 in viruses infecting *R. sinicus, R. affinis*, and *R. monoceros* that were isolated over a broad geographic range in the Yunnan (RaTG-13; Zhou et al. 2020) and Zhejiang provinces (Longquan-140, ZXC21, and ZC45; Lin et al. 2017; Hu et al. 2018), which are each ∼1,000 km away from Hong Kong/Guangdong, and ∼2,000 km apart from each other. Overall, these findings indicate that viruses carrying SARS-CoV-2-like FC sequences have been circulating now for at least 14 years, over an extended geographical area, in viruses infecting multiple species of horseshoe bats. The fact that the pangolin CoVs identified in 2020 (Zhang, Wu, and Zhang 2020) also bear a SARS-CoV-2 like FC (see Fig. 7A) further supports this conclusion, indicating a widespread circulation of this fusion module in viruses infecting mammalian taxa other than Chiroptera.

Previous molecular clock-based studies (Lau et al. 2010; Fig. 7B) date the divergence between the HKU3 virus clade, bearing a SARS-CoV-2-like FC, and the SARS-CoV-1 clade to the early ‘70s, which corresponds to the bifurcation within clade-3 in the phylogenetic tree by Lu et al. (2020; highlighted in green in Fig. 7A–B). Moreover, the latter tree also shows that the divergence between clade-3 and the SARS-CoV-2-like viruses belonging to clade-2 is clearly anterior to this date. These observations together strongly indicate that the origin of the SARS-CoV-2 FC1 sequence may lie more than 50 years ago, and that this fusion machinery may have spread to different Sarbecovirus clades by recombination, which is very frequent in the CoV spike gene (Bobay, O’Donnell, and Ochman 2020; see *Discussion*).

The combination of the results of our analyses and of previous evolutionary studies of CoVs indicates that the SARS-CoV-2 FC module originated certainly more than 15 years ago and likely has been circulating across Sarbecoviruses for more than 50 years.

### 2.8 A mutational hotspot in the SARS-CoV-2 FC during the 2019–2021 Covid-19 pandemic

**Figure 9.**
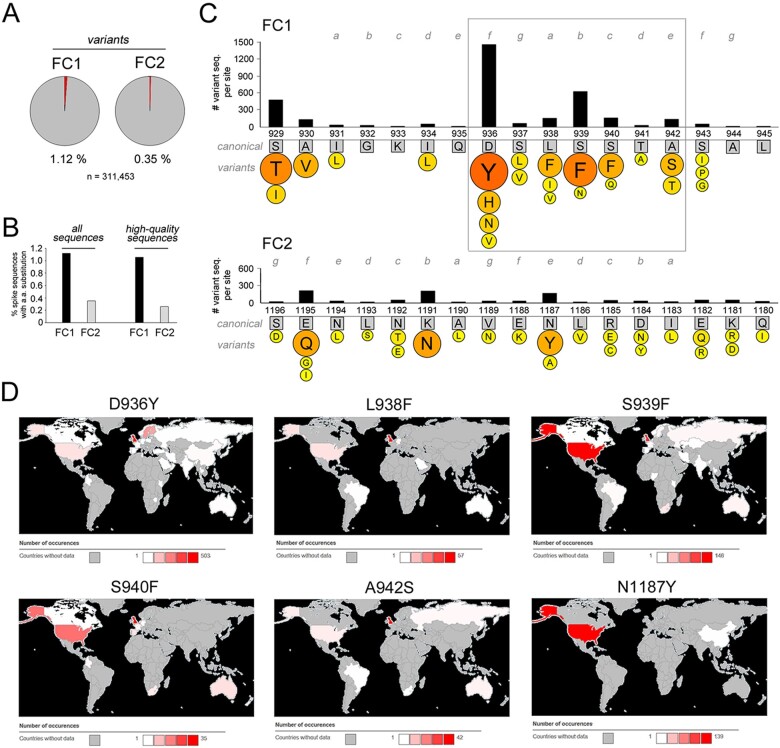
Evolution of the SARS-CoV-2 FC in the ongoing human Covid-19 pandemic. (A) Pie charts of the proportion (%) of SARS-CoV-2 sequences from the human Covid-19 pandemic, as derived from GISAID, bearing at least one amino acid substitution in the FC1 or FC2 regions in comparison with the canonical spike protein sequence. (B) Bar graphs showing the proportion (%) of SARS-CoV-2 sequences bearing at least one amino acid substitution in either the FC1 or FC2 regions, when considering all the GISAID sequences (*left graph*, as in panel A) or when excluding lower-quality sequences (*right graph*). Note how in both instances the FC1 region is more prone to amino acid substitutions. (C) Schematic representation of the sequence variability of the SARS-CoV-2 FC1 and FC2 sequences in the human pandemic. The canonical FC1 and FC2 residues are indicated within *gray squares* and numbered as in the spike protein primary sequence. The CC heptad register is highlighted in *gray*. The *bar graphs* above the primary FC1 and FC2 sequences indicate the absolute number of viral sequences bearing any amino acid substitution at each site. The *colored circles* below each canonical amino acid report the amino acids more frequently replacing the canonical ones. The size of the circles and their color, from *yellow* to *red*, are proportional to the number of sequences bearing the indicated amino acids. At each position, only amino acids found in more than 10 sequences are reported. The *box* highlights the apparent mutational hotspot in the FC1 region. (D) Geographical diffusion of the indicated SARS-CoV-2 FC1/2 variants, as derived from CoVsurver based on GISAID data. The colors of the different areas indicate the local number of sequences in which a given variant was detected, ranging from *white*, i.e. the variant was detected only once, to *red*, i.e. the variant was detected at the highest relative level, in a number of sequences indicated in each legend near the *red square*. Areas colored in *gray* are those from which the given variant was not detected or for which information was not available.

Finally, we studied the evolution of the SARS-CoV-2 FC in the ongoing human pandemic by examining the >300,000 sequences available in the GISAID database (see *Methods and*  Supplementary Table 1) that contained the entire spike protein sequence. Specifically, we identified all the sequences bearing amino acid variants in FC1 or FC2 and analyzed qualitatively and quantitatively the substitution patterns at the different sequence positions (Fig. 9). This analysis revealed, first, that amino acid substitutions were ∼3 times more frequent in FC1 (1.12 per cent of the sequences) than in FC2 (0.35 per cent), a difference that persisted even when removing from the analysis lower quality sequences containing undefined residues (‘X’; Fig. 9A–B). Interestingly, this difference parallels what observed in the evolution of Sarbecovirus, in which the FC2 sequence is conserved despite considerable FC1 variation (Fig. 8B; Supplementary Fig. S6). Furthermore, it is in agreement with early observations in the SARS-CoV-2 pandemic (Oliva et al. 2021). Taken together, these observations suggest that the FC2 sequence is subject to much stronger evolutionary constraints than FC1.

Remarkably, most of the current amino acid sequence variability in SARS-CoV-2 FC1 is concentrated in a seven residue mutational hotspot (a.a. 936–942) which encompasses four of the eight positions that in the canonical sequence differ from the SARS-CoV-1 FC1 (Fig. 9C). Another site of relatively high variability involves a.a. 929–930, whereas other FC1 positions have generally low levels of variability. The FC2 variability involves essentially only three positions, i.e. 1187, 1191, and 1195, the first of which is structurally at the same level of the FC1 936–942 hotspot in the post-fusion structure.

When we analyzed the most frequent amino acid substitutions in the FC, it was striking to observe that the residues replacing most frequently the endogenous one in the 936–942 FC1 hotspot, or in the facing FC2 position 1187, are aromatic ones, namely tyrosine (Y) and phenylalanine (F). Indeed, this more variable region corresponds to a loop in the prefusion structure that becomes α-helical when the post-fusion structure assembles (Oliva et al. 2021). Thus, the recurring aromatic substitutions in this region may modulate the transition between the pre- and post-fusion states by altering the energy barrier for the conformational change. F and Y residues can have an important structural impact on CC assemblies and Y, as in the D936Y mutant, can pack at the interface between the CC core and its solvent exposed surface (Rhys et al. 2021). Remarkably from our perspective, the L938F substitution further increases local heptad stereotypy by inserting at position *a* of the heptad register an F residue matching F927 and F970, upstream and downstream, respectively. The same pattern of heptad-spaced F residues is present in the MERS-CoV FC, which also contains a fourth F in position *a* (Fig. 2A), thus suggesting that these aromatic residues must serve an important structural role. This interpretation is strongly supported by the fact that the combination of F in *a* and aliphatic residues in *d* is known to favor the formation of antiparallel CC hexamers (Spencer and Hochbaum 2016). Other notable substitutions from the standpoint of our analysis are the A942S and the K1191N which further increase the S/Q ratio within the FC, and S929T and E1195Q that tend instead to reduce it. Finally, the S943P and S943G variants, although relatively rare, fall at the junction between the loop and the already helical portion of the FC1 sequence in the pre-fusion structure and may thus have a strong impact on the post-fusion assembly given their known helix-destabilizing and/or bending potential (e.g. Fiumara et al. 2010).

It is interesting that several of these substitutions, especially those involving aromatic residues in the FC1 hotspot, have reached a virtually worldwide distribution as their presence has been detected in 4–5 continents (Fig. 9D). These findings indicate that these variants are viable and highlight ongoing trends in the structural and functional evolution of the SARS-CoV-2 fusion machinery.

## 3. Discussion

The results of our positional and compositional analyses uncover previously unrecognized features of the SARS-CoV-2 CC-based fusion machinery, i.e. enhanced HR stereotypy and compositional drift, also defining novel analytical parameters, i.e. the heptad stereotypy index and the S/Q ratio, illuminating general features of class I FPs in viruses ranging from RSV to HIV and EBOV. Our findings may help rationalize, in quantitative and evolutionary terms, the empirically observed differential structural stability of the SARS-CoV-1 and -2 FCs, as shown in circular dichroism analyses by Zhu et al. (2020), which can only arise from the clustered FC1 changes that differentiate SARS-CoV-1 and -2. Finally, our evolutionary analyses show that the fusion module of SARS-CoV-2 is not a unique feature of this virus and its immediate precursors but is more broadly distributed within Sarbecovirus, also in viruses that are SARS-CoV-1-like otherwise, thus indicating its spread by recombination and locating its origin certainly to more than 15, and likely to more than 50, years ago.

### 3.1 Sequence stereotypy and compositional drift in conformational and functional CC switches

Our analysis focused initially on a set of nine highly clustered amino acid substitutions that differentiate the HR1 regions of SARS-CoV-1 and -2, which are identical otherwise. Notably, eight of these changes fall within the short FC1 segment which has a critical role in viral fusion, suggesting that they may impact the structure and function of the 6-HB driving the entry of viral particles into cells. This interpretation was supported by the observation that the HR1 region has a low level of variation across Sarbecovirus and displays signatures of positive selection, as revealed by the MK test. These two findings combined strongly suggest that the clustered FC1/HR1 substitutions that differentiate SARS-CoV-2 from -1 may have arisen from selective processes rather than from the mere neutral sequence divergence between these viruses.

In principle, these observed changes in the FC1/HR1 region may arise from selective pressure at both functional and antigenic levels. In this respect, it should be considered that even single-residue changes in the FC of class I FCs can have dramatic effects on viral infectivity and fusogenicity (e.g. He et al. 2007; Wang et al. 2012) and that, instead, the FC/HR regions are not among the most prominent antibody targets in the spike protein (e.g. Cerutti et al. 2021). This evidence, taken together, suggests that antigenic pressure, which cannot be ruled out, may not be the major selective determinant of FC1/HR1 evolution, which may be more driven by the selective advantage brought about by functional changes in the fusion machinery.

Based on these initial observations, we explored the impact of these substitutions on the local sequence composition in the context of the CC HRs. We found that the set of clustered amino acid substitutions within HR1 leads to an increase in sequence similarity among the FC heptads and flanking regions and considerably drifts the local amino acid composition. These substitutions concurrently reduce the local complexity of the CC-prone sequence by limiting the types of amino acids (compositional bias) and of their sequence patterns (heptad stereotypy). It is tempting to speculate that the increased heptad stereotypy that we observed within and around the SARS-CoV-2 FC, at the highest levels among the human β-CoVs, may modulate the pre-to-post-fusion structural transitions and the assembly of the FC. Indeed, even single residue substitutions within the FCs of class I FPs can have profound effects on the structural stability of the 6-HB and on the infectivity of the viral particles (e.g. He et al. 2007; Wang et al. 2012). Even some mutations occurring in the ongoing human pandemic, such as the L938F, seem to favor the further increase in the HR1/FC1 heptad stereotypy by increasing the number of F residues at heptad position *a*, somewhat paralleling what found in the homologous region of the MERS-CoV spike. Strikingly, F residues in artificial CC peptides have been shown to favor the formation of antiparallel CC hexamers (Spencer and Hochbaum 2016), i.e. the very same structure formed by post-fusion class I FPs, thus indicating that the recurrence of F residues in position *a*, also in emerging SARS-CoV-2 mutants, may have a precise structural meaning.

Interestingly, the heptad stereotypy analyses performed either taking into account, or not, the stutters in the heptad register (Parry, Fraser, and Squire 2008) were similar. This suggests that the higher internal similarity of the SARS-CoV-2 HR1 heptads that we observed may have structural relevance not only post-fusion, when the stutters become evident, but also pre-fusion, in the presence of more fragmented, not yet coiled HR1 helices (Cai et al. 2020; Oliva et al. 2021) in which the primary sequence periodicity as such may be important in some other respect.

The compositional bias that we observed toward polar residues of class I FCs in general, and toward a S/N-rich configuration in the SARS-CoV-2 FC in particular, may be related to multiple structural and functional reasons. Remarkably, polar residues forming H-bonds (‘polar layers’) have important roles in viral fusion machineries (e.g. Ji, Bracken, and Lu 2000; Suntoke and Chan 2005) and even in artificial 6-HBs that mimic viral FCs (Boyken et al. 2016). Moreover, amino acid substitutions within class I FCs can dramatically alter 6-HB stability, which correlates with changes in viral infectivity and fusogenicity (Dubay et al. 1992; Wild et al. 1994; He et al. 2007; Lu et al. 2012; Wang et al. 2012).

Polar residues may be directly relevant to the structural transitions of the HR region leading to the FC assembly. Indeed, the post-fusion 6-HB is a complex CC configuration in which FC1 residues at *e*/*g* heptad positions, that would normally be solvent-exposed in a HR1 trimer as such, interact instead with residues on the hydrophobic face of the antiparallel FC2 helices, at positions *a/d*, respectively. It is noteworthy, in this respect, that *e*/*g* positions in FC1 and flanking heptads of the SARS-CoV-2 spike and other class I FPs are frequently occupied, rather than by bulky aliphatic side chains, by either small hydrophobic residues like alanine or polar residues which can behave as ambivalent hydrophobes (Sodek et al. 1972; Akey, Malashkevich, and Kim 2001) and have α-helical and CC propensity, even in a homopolymeric form (Fiumara et al. 2010; Pelassa et al. 2014; Lilliu et al. 2018; Vaglietti and Fiumara 2021). Interestingly, some substitutions observed in the FC1 mutational hotspot during the ongoing human Covid-19 pandemic, such as A942S in *e* position, increase even further the compositional drift toward serine-richness, pushing the region between S937 and S943 toward a quasi-homopolymeric state. Polar Q/N residues in SARS-CoV-1 HR1, both upstream and downstream the FC1 region, form the lining of two central cavities hosting a chloride ion and establishing a network of H-bonds stabilizing the post-fusion conformation and affecting HR2 folding (Duquerroy et al. 2005). Thus, the enrichment in polar residues may be functional to facilitate the complex structural transitions and the post-fusion packing of residues in a 6-HB structure. Remarkably, CC domains underlying the structural/functional switches of prion-like proteins can also be highly enriched in polar residues (Fiumara et al. 2010) and SNARE complexes mediating synaptic vesicle fusion display a polar 0-layer with key functional roles that can contain Q, S, and N (Graf et al. 2005; Ayong et al. 2007). Thus, the enrichment in polar residues, which can occupy both solvent-exposed and buried position, and are therefore suitable for multiple structural contexts, may represent a more general feature of metastable, fast-switching helical/CC structural assemblies. This compositional feature adds to other similarities between CCs in viral FPs and SNARE proteins (Skehel and Wiley 1998).

Polar residues such as N, which recurs in position *d* in FC-flanking heptads of SARS-CoV-2, SARS-CoV-1, and MERS-CoV HR1, when present at CC core positions can specify oligomeric states (Gonzalez et al. 1996; Fletcher et al. 2017) and complex, artificial CC configurations can be assembled by rationally designing buried H-bond networks formed by polar residues (Boyken et al. 2016). Our findings indicate that changes in the polar residue composition of class I FCs, ranging from S/N- to Q/T-rich configurations, are associated with changes in the relative proportion of intra- *versus* inter-helical H-bonds in the post-fusion structure, which may have functional impact. Indeed, amino acid substitutions in viral class I FCs, by altering inter-helical interactions and H-bonding, can significantly reduce the structural stability of the 6-HB which correlates with an impairment in the infectivity/fusogenicity of mutant viral particles (e.g. Wang et al. 2012; He et al. 2007; Dubay et al. 1992; Wild et al. 1994; Lu et al. 2012). Notably, even in artificial systems of CC-mediated membrane fusion, the structural stability of HR peptides correlates with fusion efficiency (e.g. Zheng et al. 2013).

Our findings show that a higher S/N content predicts a higher proportion of inter-helical H-bonds in the post-fusion 6-HB of class I FPs of CoVs and other viruses. This observation was directly exemplified by the increased overall number of S/N-mediated inter-helical bonds, and by the parallel reduction in the density of the intra-helical bonds, in the central part of the SARS-CoV-2 FC in comparison with the SARS-CoV-1 FC. These observations may rationalize in structural terms the emprically observed higher thermal stability of the SARS-CoV-2 FC in comparison with the SARS-CoV-1 FC in circular dichroism experiments (Zhu et al. 2020).

The enrichment in polar residues at solvent-exposed positions may also mediate lateral interactions and oligomerization of post-fusion spikes facilitating fusion pore opening (Danieli et al. 1996).

Another functional consequence of the enrichment of polar residues in the HR/FC region may be related to post-translational modifications and antigenicity. Notably, N and S/T residues can undergo N-linked or O-linked glycosylation, respectively. Cai et al. (2020) observed five glycosylated N residues in the HR2 region, one of which (N1194) falls within the FC2 α-helix, and noted that glycans at this level would substantially shield the post-fusion structure from the immune system. This feature may be functionally relevant if spikes in post-fusion conformation, spontaneously formed independent of ACE2 receptor binding, are present on the surface of mature virions, where they may protect from the immune system, as decoys, the pre-fusion spike trimers (Cai et al. 2020).

The HR regions of CoV spike proteins contain epitopes that can elicit the production of neutralizing antibodies (Lip et al. 2006; Grifoni et al. 2020), although they are not major antigenic targets (Cerutti et al. 2021). Thus, the compositional changes shifting the relative occurrence of polar residues, such as those we observed between SARS-CoV-1 and -2, may also impact the antigenicity of the HR/FC region. Remarkably, S-rich surface antigens have also been found in bacteria and eukaryotic parasites, including virulent strains of *Streptococcus agalactiae* and *Plasmodium falciparum*, and can elicit protective antibodies (Seifert et al. 2006; Yagi et al. 2014). Thus, S-rich sequences in viral, bacterial, and eukaryotic pathogens may represent shared immunological targets with similar structural features.

### 3.2 Analysis of the SARS-CoV-2 spike illuminates general features of class I FPs: S/Q ratio and alternate post-fusion layering

The enhanced heptad stereotypy and compositional drift of the SARS-CoV-2 FC are accompanied by a dramatic increase in the serine-to-glutamine (S/Q) ratio.

A comparative analysis revealed that SARS-CoV-2 ranks among viruses with the highest S/Q ratio in the FC, which have respiratory tropism and strong syncytium-forming capacity (RSV, NDV; Li et al. 1998; Morton et al. 2003; Tian et al. 2013). The SARS-CoV-1 and other human CoVs are known to be syncytiogenic (e.g. Kuiken et al. 2003). Strinkingly, however, the SARS-CoV-2 spike protein is significantly more fusogenic and less dependent on protease activation than the SARS-CoV-1 spike, and both these features may critically enhance the SARS-CoV-2 infectivity, together with an higher affinity for the ACE2 receptor (Musarrat et al. 2020; Ou et al. 2020; Xia et al. 2020). Moreover, the formation of syncitia of pneumocytes is a paramount histopathological feature of SARS-CoV-2 affected lungs (Bussani et al. 2020) and can even be induced by viral particles fusing with adjacent cells (Theuerkauf et al. 2021).

Thus, it is possible that the higher proportion of S/N residues in class I FCs may be associated with a higher fusogenic and syncytiogenic potential, perhaps by increasing the number of the interhelical H-bonds, in comparison with more Q/T-rich FCs, as indicated by our analyses. This possibility is consistent with observations that mutations in the FC/HRs of class I fusion machineries can regulate syncytium formation (McGinnes et al. 2001; Plemper and Compans 2003, 2003; Dubois et al. 2017) and that interhelical interactions mediated by polar residues in the FC modulate the HIV fusogenic potential, and ultimately its infectivity (Suntoke and Chan 2005). In particular, Dubois et al. (2017) indicate that enhanced fusogenicity can be induced by a few key residues in the HRA domain (i.e. the structural equivalent to the SARS-CoV-2 FC1), likely due to the stabilization of the post-fusion structure. Notably, the 6-HB formed by SARS-CoV-2-derived HR1/HR2 peptides *in vitro* is considerably more stable than the one formed by SARS-CoV-1-derived peptides (Zhu et al. 2020) and this difference can only be caused by the nine clustered mutations in HR1 that differentiate the two viruses, four of which (44 per cent) are substitutions to serine. These pieces of evidence combined support the hypothesis that the serine-richness of the SARS-CoV-2, RSV, and NDV may be related to their higher syncytiogenic potential. However, this hypothesis arising from our *in silico* analyses will need to be tested experimentally.

Besides the enrichment of S residues in the FC region, we observed a striking alternate S/Q layering along the SARS-CoV-2 post-fusion spike structure. Such a layering is modified in the post-fusion spike structures of SARS-CoV-1 and MERS-CoV, which still present alternate S- and Q-rich subregions but with less defined and/or shifted boundaries. Remarkably, such alternate layering of polar regions can also be observed in the post-fusion structures of viruses which are not part of *Coronaviridae*, such as the NDV (*Paramyxoviridae*), thus representing a recurring, unappreciated feature of the class I fusion machineries. These observations strongly suggest that the recurrent S/Q segregation patterns along the post-fusion class I FP structures may have a precise functional meaning that will have to be assessed experimentally. An attractive possibility suggested by our analyses is that the alternate layering of S- and Q-rich areas may be related to alternate patterns of inter- and intra-helical H-bonds, ultimately contributing to the pre-to-post-fusion structural transitions and to the stabilization of the post-fusion 6-HB. As structural stability is directly related to the efficiency of CC-mediated membrane fusion, even in artificial systems (Zheng et al. 2013), the functional meaning of the different S/Q layering patterns may be related to the regulation of viral infectivity.

These hypotheses on the possible functional and pathobiological consequences of the different S/Q ratios will have to be validated experimentally in structural studies inspired by our *in silico* analyses.

### 3.3 Evolutionary origin and spread of a S-rich FC configuration in the Sarbecovirus subgenus

The origin of SARS-CoV-2 is still debated. Multiple lines of evidence indicate that the virus might have originated by recombination events between viruses infecting mammals, such as bats and pangolins, with a possible adaptation phase in humans after the original spillover events (e.g. Andersen et al. 2020). The CoV spike is particularly prone to recombination events and several studies traced the origin of the RBD and furin cleavage site in SARS-CoV-2 (Liu et al. 2020; Zhang, Wu, and Zhang 2020), whereas the origin of the FC of this virus had not been investigated in detail.

We found that the SARS-CoV-2 FC is present not only in phylogenetically related viruses isolated in bats (RaTG13) and pangolins, which were soon identified as its direct precursors (Zhang, Wu, and Zhang 2020), but also in a series of more distantly related Sarbecoviruses, whose spike proteins are SARS-CoV-1-like in any other respect. These viruses have been repeatedly identified since 2005 and evolutionary analyses trace the likely origin of their SARS-CoV-2-like FC to more than 50 years ago (Lau et al. 2010), suggesting that recombination facilitated the spread of this viral fusion module across viruses in different branches of the Sarbecovirus phylogenetic tree. Finally, the analysis of current mutational trends in >300,000 available SARS-CoV-2 genomes shows that the FC portion within HR1 is much more prone than the HR2 portion to amino acid substitutions. The HR1 substitutions tend to cluster in a hotspot of a few amino acids that are frequently replaced by aromatic residues or serine, thus further increasing the local frequency of this latter amino acid.

The origin and presence of SARS-CoV-2-like FCs in both the SARS-related and SARS-CoV-2-related clades of the Sarbecovirus subgenus can be explained in principle by different evolutionary dynamics. First, the same S-rich SARS-CoV-2-like FC may have arisen by convergent evolution multiple times. While this possibility cannot be ruled out, it seems fairly unlikely given the absolute identity of the FC1 amino acid sequences of SARS-CoV-2 and viruses such as HKU3-7 which are SARS-CoV-1-like otherwise. Second, it could be assumed that the common ancestor of both the SARS-CoV-1-like and the SARS-CoV-2-like viruses had originally a SARS-CoV-2-like FC1 and that SARS-CoV-1-like viruses diverged at some point of their evolution developing a different FC1 sequence. However, the presence of more SARS-CoV-1-like FCs in ancestral Sarbecoviruses such as BtKY72 and BM48-31 (clade-1 in the tree by Lu et al. 2020) suggests rather the opposite scenario, with a common Sarbecovirus ancestor bearing a more SARS-CoV-1-like FC1. A fourth, and perhaps more parsimonious explanation, considering the recombination proneness of the β-CoV spike protein gene (Bobay, O’Donnell, and Ochman 2020), is that the S-rich FC originated in a common ancestor of the SARS-CoV-2-like clade and spread by recombination to a common ancestor of the Sarbecovirus clade encompassing the Lonquan-140 virus and the HKU3 virus clade. This recombination event is plausibly not recent as the nucleotide similarity between the FC1-encoding region of SARS-CoV-2 and viruses like HKU3-1, HKU3-7, and Longquan-140 is lower than the amino acid similarity (see Fig. 8B and Supplementary Fig. S6).

The substantial conservation of two alternative FC modules across Sarbecoviruses suggests that these two configurations may be highly functional and subject to considerable selective pressure, as also observed for the MERS-CoV FC sequences (Forni et al. 2015). The two Sarbecovirus FC configurations may grant different fusion efficiency and ultimately viral infectivity. These differences may explain some of the epidemiological differences between SARS and Covid-19. Moreover, this knowledge will also be important in tracing the diffusion of SARS-CoV-1-like and SARS-CoV-2-like viruses in surveillance programs (e.g. Rizzo et al. 2017) that will be fundamental in the post-Covid-19 era to monitor and prevent future pandemics.

In conclusion, our findings identify defining positional and compositional features, such as the S/Q ratio and alternate layering, of the SARS-CoV-2 fusion machinery that illuminate more general aspects in the evolution of class I FPs, tracing at the same time the long, ongoing, phylogenetic history of this essential functional module of the spike protein within the Sarbecovirus subgenus. The long evolutionary history of the SARS-CoV-2 FC is continuing in the ongoing pandemic as a consequence of mutations that may further increase the viral fusion capacity and infectivity. Besides their relevance to our understanding of SARS-CoV-2 biology, these results offer a novel, general classification framework for class I FPs, based on their content and spatial segregation of polar residues, which may facilitate the structure-guided development of specific viral fusion inhibitors for SARS-CoV-2 and other emerging pathogens.

## 4. Methods

### 4.1 Databases, protein structures, sequences, and domain boundaries

Protein primary sequences of the FPs of CoV and other viruses of interest were obtained from Uniprot: P0DTC2 (Spike glycoprotein, Severe acute respiratory syndrome coronavirus 2, SARS-CoV-2), P59594 (Spike glycoprotein, Human SARS coronavirus, SARS-CoV-1), K9N5Q8 (Spike glycoprotein, Middle East respiratory syndrome-related coronavirus, MERS-CoV), Q5MQD0 (Spike glycoprotein, Human coronavirus HKU1, HCoV-HKU1), P36334 (Spike glycoprotein, Human coronavirus OC43, HCoV-OC43), Q6Q1S2 (Spike glycoprotein, Human coronavirus NL63, HCoV-NL63), P15423 (Spike glycoprotein, Human coronavirus 229E, HCoV-229E), P03420 (Fusion glycoprotein F0, Human respiratory syncytial virus A, RSV), P35936 (Fusion glycoprotein F0, Newcastle disease virus, NDV), O89342 (Fusion glycoprotein F0, Hendra virus, HeN), Q9IH63 (Fusion glycoprotein F0, Nipah virus, NiV), P33481 (Fusion glycoprotein F0, Mumps virus, MuV), P05877 (Envelope glycoprotein gp160, Human immunodeficiency virus type 1, HIV), P17332 (Pre-glycoprotein polyprotein GP complex, Lassa virus, LASV), Q05320 (Envelope glycoprotein, Zaire ebolavirus, EBOV), P03454 (Hemagglutinin, Influenza A virus).

Post-fusion crystal structures of spike proteins of interest for the H-bonds analyses were derived from the Protein Data Bank (PDB; https://www.rcsb.org; Berman et al. 2002). The PDB IDs are: 6LXT (SARS-CoV-2), 1WYY and 2BEZ (SARS-CoV-1), 4NJL (MERS-CoV virus), 2IEQ (HCoV-NL63 virus), 5YL9 (HCoV-229E virus), 1G2C (RSV), 3MAW (NDV), 1WP8 (HeV), 1WP7 (NiV), 2FYZ (Mumps virus), 1ENV (HIV), 5OMI (LASV), 2EBO (EBOV), 1HTM (Influenza virus). These structures have been described in: (Bullough et al. 1994; Weissenhorn et al. 1997; Malashkevich et al. 1999; Zhao et al. 2000; Supekar et al. 2004; Duquerroy et al. 2005; Liu et al. 2006; Zheng et al. 2006; Lou et al. 2006; Swanson et al. 2010; Lu et al. 2014: Yan et al. 2018; Shulman et al. 2019; Xia et al. 2020). We also used the cryo-EM pre- and post-fusion SARS-CoV-2 spike structures by Cai et al. (2020; PDB: 6XR8 and 6XRA, respectively) to generate some images presented in the figures.

For SARS-CoV-2 FC mutation analyses, spike protein sequences were downloaded from the GISAID database (https://www.gisaid.org; Elbe and Buckland-Merrett (2017); see the list of contributors in Supplementary Table 1).

For comparative analyses of FC amino acid substitutions in human and animal CoVs, we downloaded sequences of interest, as listed by the Zhang, Wu, and Zhang (2020), Lu et al. (2020), and Lau et al. (2010) (see *Results*), from the NCBI Virus database (Hatcher et al. 2017; https://www.ncbi.nlm.nih.gov/labs/virus/vssi/#/sars-cov-2).

In the analyses that we performed, the SARS-CoV-2 spike protein domain boundaries were defined as indicated in Uniprot, in relation to the reference sequence P0DTC2 (1,273 residues), or based on the manual inspection of spike protein structures reported in the PDB database (6XRA, 6LXT), as follows: N-terminal region encompassing the S1 domain, residues 1–527; heptad repeat 1 (HR1), residues 913–1034; FC portion within HR1 (FC1), residues 929–945; heptad repeat 2 (HR2), residues 1163–1202; FC portion within HR2 (FC2), residues 1180–1196; HR2 + C-terminal region (HR2+), residues 1163–1273. The corresponding regions in other CoV spike protein sequences were identified based on the alignment with the SARS-CoV-2 sequence. The FC1 and FC2 boundaries in the class I FPs of CoV and non-CoV pathogens were defined, based on the manual inspection of post-fusion PDB structures (see *Results*), as the HR1 and HR2 tracts with helical structure facing each other in the post-fusion 6-HB.

### 4.2 Shannon entropy analysis

The analysis of evolutionary sequence variability was performed on amino acid sequence alignments of spike proteins of viral clades of interest by calculating the Shannon entropy for each alignment position using the *Entropy-one* tool, available at the Los Alamos National Laboratory website (www.hiv.lanl.gov/content/sequence/ENTROPY/entropy.html), using a 5-residue window.

We used three sequence alignments of representative spike protein sequences of α/β-CoVs and Sarbecovirus included in the phylogenetic tree by Zhang, Wu, and Zhang (2020; as reported in Supplementary Fig. S5), and of β-CoVs, derived from the phylogenetic tree by Lu et al. (2020, as reported in Fig. 7A),

### 4.3 MK test

The MK test (McDonald and Kreitman 1991) on alignments comprising the HR1 or HR2+ nucleotide sequences to compare intra-clade and inter-clade sequence variation (e.g. Xu et al. 2018) of these two spike regions in SARS-CoV-2-like viruses *versus* SARS-CoV-1 was performed using the DnaSP6 package (Rozas et al. 2017), which calculates the NI and performs statistical significance tests (Fisher’s exact and G tests). The NCBI sequence IDs of the spike CDS sequences that were aligned using ClustalOmega (https://www.ebi.ac.uk/Tools/msa/clustalo/) are: AAP51227.1, AVP78031.1, AVP78042.1, QHD43416.1, QHR63300.2, QIG55945.1. The MK test was performed for HR1 on the alignment region corresponding to nucleotides from 2737 to 3102 (i.e. 366 nucleotides) of the SARS-CoV-2 spike CDS. The nucleotide sequence encoding HR2 was too short to give statistically meaningful results with the MK test. Thus, we analyzed the HR2 region together with the flanking C-terminal region of the spike (up to the stop codon), which has similarly low evolutionary variability levels (see Fig. 1D). This longer, HR2-encompassing region (*HR2+*) corresponds in the alignment used for the MK test to nucleotides from 3487 to 3819 (i.e. 333 nucleotides) of the SARS-CoV-2 spike CDS. The test was performed after ruling out substitution saturation in the sequence alignments of spike protein sequences of interest using the DAMBE software package (Xia et al. 2003, 2017) using the method by Xia and Lemey (2009). In this analysis, the software preliminarily calculates the proportion of invariant sites. Then it calculates an index of observed substitution saturation (*Iss*) and compares it with a critical index (*Iss-c*), representing the threshold for significant saturation (Xia et al. 2003). Significant saturation is ruled out when *Iss* is significantly smaller than *Iss-c*.

### 4.4 Heptad stereotypy analyses

The degree of heptad similarity in the FC1 and flanking HR1 regions in SARS-CoV-2 was calculated by counting, for each pair of heptads, the number of exact residue matches at each one of the heptad register positions. We analyzed in this manner the SARS-CoV-2 HR1 spike region comprised between the two 4-residue stutters (Cai et al. 2020; Walls et al. 2020), together with two flanking heptads, one upstream and one downstream (see Fig. 2A). These heptads were numbered from 1 to 9, where the second one and the eight one correspond to the two stutters (i.e. ‘FNSA’ and ‘LSSN’, respectively). The corresponding HR1 regions of the closely related SARS-CoV-1 and MERS-CoV, as determined based on a primary sequence alignment, were analyzed in the same manner. As an additional measure of heptad stereotypy, for each virus, we also counted separately the relative proportions of residue matches found in heptad pairs with minimal similarity (none or one match) and from heptad pairs with higher similarity (two or more matches). The same analyses were also performed not taking into account the presence of the stutters in the heptad register, i.e. on the same HR1 regions with a few additional upstream and downstream residues (see Supplementary Fig. S1C). The heptad-to-heptad similarity scores were also used to generate data networks using Cytoscape (see *below*) visually summarizing the results of these analyses (see Fig. 2B and Supplementary Fig. S1D).

We also performed stereotypy analyses by studying amino acid recurrence at the non-*a*/*d* positions of the heptads 1–9 (as indicated in Fig. 2A). We did not include *a*/*d* heptad positions in this analysis as they are, almost by definition, stereotypical in CC sequences, i.e. they are typically occupied by leucine or other aliphatic residues (Parry, Fraser, and Squire 2008). In these analyses, we calculated a ‘stereotypy index’ to measure the diversity of amino acid composition at each heptad position in each virus. In the nine analyzed heptads, we counted for each virus (1) the number of heptads in which the given position was occupied by amino acids recurring more than once across the nine heptads at the same position (*r*) and (2) the number of heptads occupied instead by amino acid residues occurring only once (*o*). The stereotypy index was calculated as the *r_x_*/*o_x_* ratio at each position (‘*x*’) for each virus. We also calculated the overall stereotypy index for the *b*/*c*/*e*/*f*/*g* positions as (*r_b_ *+ *r_c_ *+ *r_e_ *+ *r_f_ *+ *r_g_*)/(*o_b_ *+ *o_c_ *+ *o_e_ *+ *o_f_ *+ *o_g_*). While this was not the case of our analysis, in cases in which at one of the positions *o_x_* is equal to 0, then the stereotypy index can be approximated as *r_x_*/(*o_x_ *+ 0.5) for all positions. The statistical significance of the difference between the stereotypy index with or without the contribution of the FC1 heptads was assessed using the *χ*^2^ test on a contingency table including the values of (*r_b_ *+ *r_c_ *+ *r_e_ *+ *r_f_ *+ *r_g_*) and (*o_b_ *+ *o_c_ *+ *o_e_ *+ *o_f_ *+ *o_g_*) calculated either with (+) or without (−) the FC1 heptads.

### 4.5 Amino acid frequency plots

The local frequency of amino acids of interest along the primary sequence of class I FPs was calculated using ad hoc Perl scripts. These scripts calculated the percent occurrence of a given amino acid in a 21-residue sliding window centered on each residue along the entire primary sequence of a given protein. These values were normalized to the overall percent occurrence of the same amino acid in the entire primary sequence of the protein. Plots of these normalized values along the primary sequence of the protein were used to identify local peaks of amino acid overrepresentation.

### 4.6 Fusion core composition

The boundaries of the 6-HB FCs in the primary sequences of FPs of interest were manually determined by visual inspection of the relevant PDB crystal structures. As a general criterion, we included as part of the FC those residues in a helical conformation in the overlap region between HR1 and HR2 in the 6-HB post-fusion structures. An ad hoc Perl script was used to calculate the absolute and percent occurrence of amino acids of interest in either the HR1- and HR2-derived parts of the FC, i.e. FC1 and FC2, respectively, as well as in the FC overall (FC1 + FC2). The influenza virus was not included in the S/Q ratio analysis as its FC contains in the helical portion only single S and Q residues, making it non-representative in quantitative terms for this analysis.

### 4.7 Hydrogen bond analysis

To analyze the relative proportion of intra- *versus* inter-helical bonds in the post-fusion 6-HBs of viruses of interest, the relevant PDB structures were sequentially processed and analyzed using Yasara (Land and Humble 2018) and UCSF Chimera (Pettersen et al. 2004) software. Briefly, the six helices of each 6-HB were renamed from *A* to *F* using Yasara and the H-bonds were predicted using Chimera with default parameters. A Perl script was then used to count the number of intra- and inter-helical H-bonds. For this analysis, only intra-/inter-helical H-bonds involving at least one amino acid located within the FC boundaries were counted.

### 4.8 Analysis of the evolutionary origin of the SARS-CoV-2 FC

To study the evolutionary origin of the SARS-CoV-2 FC, we compared the FC1 sequence with that of other CoVs in reference evolutionary trees encompassing α/β-CoVs (Zhang, Wu, and Zhang 2020), β-CoVs (Lu et al. 2020), and Sarbecovirus (Lau et al. 2010). Toward this aim, we first obtained the primary amino acid sequences of the spike proteins of the viruses comprised in these trees from the NCBI Virus database (https://www.ncbi.nlm.nih.gov/labs/virus/vssi/#/sars-cov-2; Hatcher et al. 2017). Then, for each tree, we aligned the spike protein sequences using Clustal Omega (Sievers et al. 2011). Finally, we counted for each virus sequence in the alignment the number of residue matches, with either the SARS-CoV-1, or -2, at the nine positions that distinguish the SARS-CoV-2 FC1/HR1 (see Fig. 1B) from those of SARS-CoV-1. We also calculated for each virus the difference (Δ) between the number of matches with the SARS-CoV-2 and the SARS-CoV-1 FC1/HR1 sequences. Both the absolute number of matches and the Δ value allowed us to categorize the FC1/HR1 region of the CoVs that were analyzed as SARS-CoV-2-like (Δ = 9) or SARS-CoV-1-like like (Δ = −9), with intermediate degrees. To compare the amino acid sequence similarity between the entire spike proteins of viruses of interest and the SARS-CoV-2 one, after their alignment with Clustal Omega (see *below*), we used the Recombination Analysis Tool (RAT) software (Etherington, Dicks, and Roberts 2005), using a ten-residue window, every two residues. We also calculated the similarity scores between the entire spike protein sequences, or their HR1/FC1 and FC2 fragments, of viruses of interest with that of SARS-CoV-2, using a distance matrix generated by Clustal Omega from aligned sequences. A similar approach was used for the spike protein-coding nucleotide sequences. The host species/taxa of CoVs of interest indicated in Figures 7–8 are abbreviated as follows: *Aselliscus stoliczkanus*: Ase sto; *Chaerephon plicatus*: Cae Pli; Chiroptera: Chiropt; *Erinaceus europaeus*: Eri eur; *Hipposideros pratti*: Hip pra; *Homo sapiens*: Hom sap; *Mus musculus*: Mus mus; *Paradoxurus hermaphroditus*: Par her; *Pipistrellus abramus*: Pip abr; *Rattus norvegicus*: Rat nor; *Rhinolophus affinis*: Rhi aff; *Rhinolophus blasii*: Rhi bla; *Rhinolophus ferrumequinum*: Rhi fer; *Rhinolophus macrotis*: Rhi mac; *Rhinolophus pusillus*: Rhi pus; *Rhinolophus sinicus*: Rhi sin; *Rousettus leschenaultii*: Rou les; *Tylonycteris pachypus*: Tyl pac; Viverridae: Viverr/Civet.

### 4.9 Analysis of SARS-CoV-2 FC variants in the human Covid-19 pandemic

A total of 312,040 SARS-CoV-2 spike protein sequences in FASTA format were downloaded from the GISAID database (Elbe and Buckland-Merrett 2017) on 5 January 2021 (see the list of contributors in Supplementary Table 1). From this dataset, to limit the number of lower-quality sequences, we excluded sequences shorter than 1,200 amino acids (0.18 per cent of the total; the length of the canonical spike protein sequence is 1,273 amino acids) and, in some analyses, those sequences with undetermined residues (‘X’; 24.88 per cent). These spike protein sequences were analyzed using ad hoc Perl scripts. First, we identified subsets of protein sequences with any amino acid sequence mismatch with the canonical SARS-CoV-2 spike protein sequence (Uniprot ID P0DTC2) in the FC1 or FC2 regions. Then, we further analyzed these subsets of variant spike proteins and quantified, for each one of the FC1 and FC2 amino acid positions, the number of sequences bearing residues alternative to those in the canonical sequence. Finally, to define the range of sequence variability, we quantified the relative proportion of the different amino acids replacing the canonical ones at each one of the FC1 and FC2 positions.

### 4.10 Phylogeography

The geographical location and date of collection of samples/specimens for the isolation of bat viruses of interest were obtained from the relevant NCBI records associated with the corresponding nucleotide/amino acid sequences of the spike protein and/or from the related publications. Geographical maps representing the global distribution of SARS-CoV-2 variants of interest originated in the human Covid-19 pandemic were obtained from CoVSurver (https://corona.bii.a-star.edu.sg/) based on GISAID data and graphically modified.

### 4.11 Software and graphics

Analyses of heptad stereotypy, amino acid relative frequencies, FC amino acid composition, FC residue matches, and GISAID sequence analyses were performed using ad hoc Perl scripts. Nucleotide and amino acid sequence alignments were obtained using Clustal Omega (Madeira et al. 2019). Cytoscape (Shannon et al. 2003) was used to generate graph representations of heptad stereotypy, H-bond patterning, and HR1/HR2 mutations observed in the human pandemic. Sequence similarity plots of spike protein amino acid sequences were obtained using RAT by Etherington, Dicks, and Roberts (2005). MegaX (Kumar et al. 2018) was used to generate unscaled trees of SARS-CoV-1-like and SARS-CoV-2-like viruses. Molecular graphics of PDB-derived structures were obtained using UCSF Chimera (Pettersen et al. 2004). H-bond analyses were performed using Yasara (Land and Humble 2018) and UCSF Chimera. Maps of the geographical distribution of SARS-CoV-2 variants of interest were obtained from CoVSurver (https://corona.bii.a-star.edu.sg/) based on GISAID data and graphically modified. The bat silhouette by Yan Wong for Rhinolophus spp. in Fig. 8 was obtained from PhyloPic.org (CC0 1.0 licence) and modified. The geographical maps in Fig. 8 are modified versions of two maps obtained from Vemaps.com (https://vemaps.com/china/cn-02) and d-maps.com (https://d-maps.com/carte.php?num_car=137949&lang=en). Images and figures were processed using Photoshop Elements 11 (Adobe). Excel (Microsoft) was used to analyze data and generate graphs.

### 4.12 Statistics

Excel (Microsoft) and Statistica (Tibco) were used to perform data analyses and statistical comparisons as reported in the Results section. In all instances, a *p* < 0.05 was considered as statistically significant.

## Supplementary Material

veab097_Supp

## Data Availability

Data are available on request to the corresponding author.
