# Supplementary material for: Heptad stereotypy, S/Q layering, and remote origin of the SARS-CoV-2 fusion core
Source: Virus Evol. 2021 Dec 15;7(2):veab097. doi: 10.1093/ve/veab097 (PMC8754743; doi:10.1093/ve/veab097)
Supplement: veab097_Supp [file veab097_supp.zip › Marchetti et al. - Supplemental Materials + Figures.pdf]

Supplemental materials

Heptad stereotypy, S/Q layering, and remote origin of the SARS-CoV-2 fusion core

Chiara Marchetti[1#], Serena Vaglietti[1#], Francesca Rizzo[2], Giovanna Di Nardo[3], Luca Colnaghi[4,5], Mirella Ghirardi[1,6], and Ferdinando Fiumara[1,6]*

[1]*Rita Levi Montalcini* Department of Neuroscience, University of Torino, 10125 Torino, Italy;
[2]Istituto Zooprofilattico Sperimentale (IZS), 10148, Torino, Italy;
[3]Department of Life Sciences and Systems Biology (DBIOS), University of Torino, 10123 Torino, Italy;
[4]Division of Neuroscience, IRCCS San Raffaele Scientific Institute, Milano, Italy;
[5]School of Medicine, Vita-Salute San Raffaele University, Milano, Italy;
[6]National Institute of Neuroscience (INN), University of Torino, 10125 Torino, Italy.

#These authors contributed equally to this work.

*Correspondence: ferdinando.fiumara@unito.it

SUPPLEMENTAL FIGURE LEGENDS

Supplemental Figure 1 – Spike protein sequence variation in α/β-CoVs and heptad stereotypy in the the SARS-CoV-2 spike protein calculated irrespective of register stutters. **A.** As in *Fig. 1D* for an alignment of α/β-CoV spike proteins of CoVs listed in the phylogenetic tree by Zhang et al., 2020 (see Suppl. Fig. 5). A one-way ANOVA revealed overall significant differences between the mean entropy across the whole spike alignment ('spike') and some of its functional domains such as the S1 region and the HRs 1 and 2 ($F_{(3,2695)} = 41.39$, $p < 0.001$). The same analysis showed that HR1 has a significantly lower entropy in comparison with the whole spike protein ($p < 0.001$, Newman-Keuls (NK) *post hoc* test), whereas the S1 region displayed a significantly higher mean entropy ($p < 0.03$). The mean entropy of the HR2 was still lower than the spike mean entropy, as in the Sarbecovirus and in the β-CoV analyses, but the difference was not statistically significant (p = 0.12). **B.** Residues at positions *e*/*g* of the heptads corresponding to the SARS-CoV-2 heptads 1-9 (see Fig. 2A) in a sequence alignment of the FC1 sequences of the seven human α/β-CoVs. The two α-CoVs (HCoV-229E and -NL63) have two additional heptads within FC1 (α_a and α_b). Note how heptad-spaced alanine residues recur repeatedly at *e*/*g* positions, particularly in the two α-CoVs and in the MERS-CoV. **C.** As in *Fig. 1D* but the stutters in the heptad register (Parry et al., 2008) observed in the crystal structure are not considered. The heptad register is based on heptad 3 within the FC. **D.** As in *Fig. 1E* without considering the two stutters in the heptad register. **E.** Sum of the residue matches at each heptad position in all the pairwise heptad-to-heptad comparisons, for the indicated viruses calculated when considering stutters (see *Fig. 1D*). **F.** Proportion (%) of the residue matches at each heptad position in all the pairwise heptad-to-heptad comparisons, for the indicated viruses, found in heptad pairs with minimal similarity (only 1 match, *red bars*) and in heptad pairs with higher similarity (2 or more matches, *cyan bars*), taking stutters into account (see *Fig. 1D*). **G-H.** As in *panels C-D*, respectively, without considering stutters.

Supplemental Figure 2 – S-rich, Q-rich and mixed FCs and alternate S/Q layering in post-fusion spike protein structures of human α-CoVs.

Ribbon structure images as in *Fig. 2,* for the two human α-CoVs viruses, showing the overall post-fusion structure (*upper panel*). The *lower panel* shows the same structure but with only Q (*red*) and S (*green*) residues visible.

Supplemental Figure 3 – S-rich, Q-rich, and mixed FCs and alternate S/Q layering in post-fusion spike protein structures of viruses with class I FPs.

Ribbon structure images as in *Fig. 2,* for the non-CoV human viruses with class I FPs, showing the overall post-fusion structure (*upper panel*). The *lower panel* shows the same structure but with only Q (*red*) and S (*green*) residues visible.

Supplemental Figure 4 – Composition of the FCs of class I FPs and negative correlation between the occurrence of Q and N residues, and S and T residues.

A. As in *Fig. 4A* for the indicated viruses. **B.** Scatterplot representing the inverse correlation between the percent occurrence of Q and N residues in the FCs of the 7 human CoVs and other representative viruses with class I FPs. **C.** As in *panel B,* for S and T residues.

Supplemental Figure 5 – SARS-CoV-2-like and SARS-CoV-1-like FCs in α- and β-CoVs.

A. As in *Fig. 6A* for the phylogenetic tree of α- and β-CoVs from Zhang and coll. (2020), based on whole-genome sequences, with minor simplifications.

Supplemental Figure 6 –Phylogenenesis of the SARS-CoV-2 FC.

A. As in *Fig. 7B* for the nucleotide sequences.

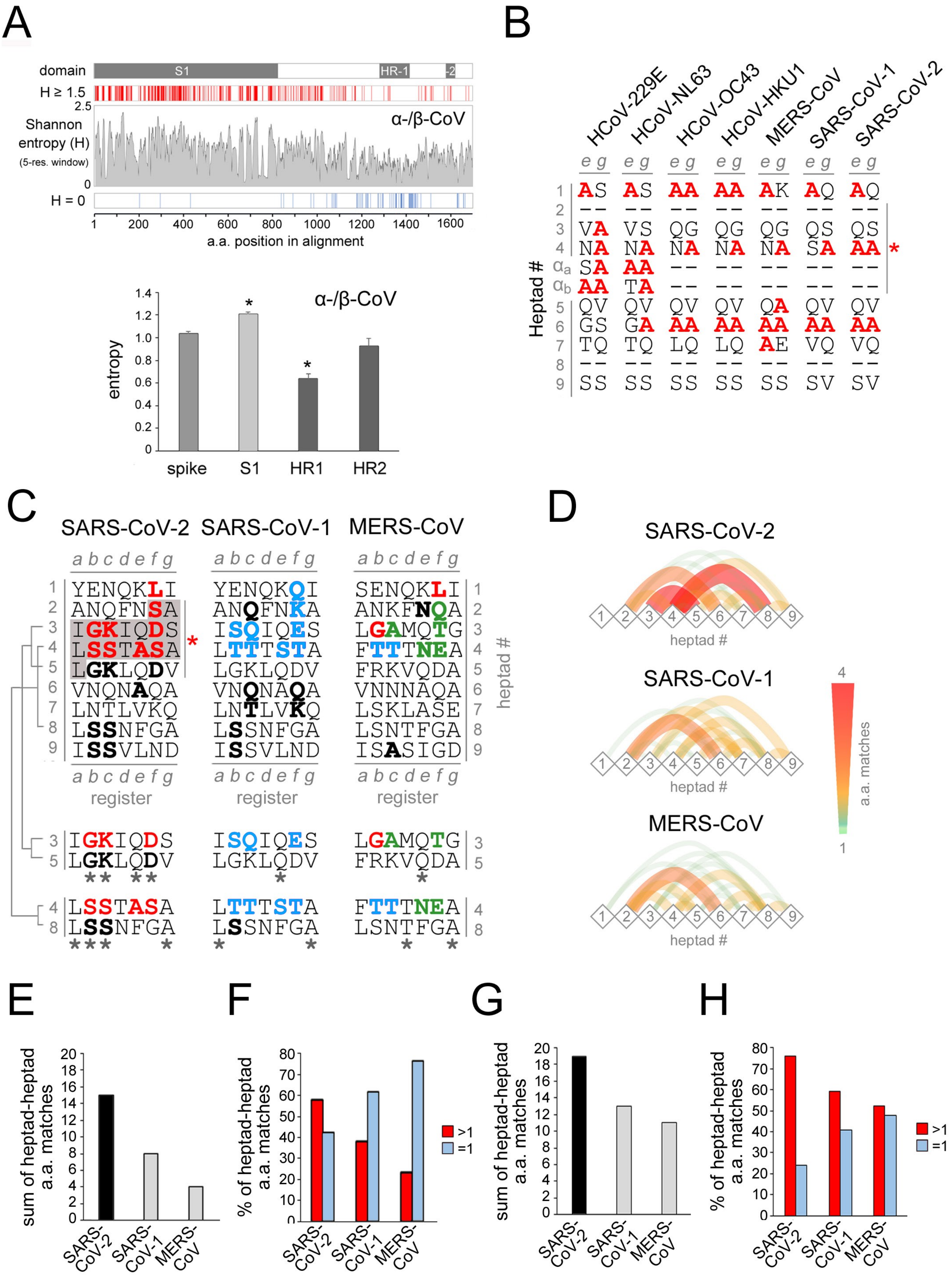

Suppl. Figure 1

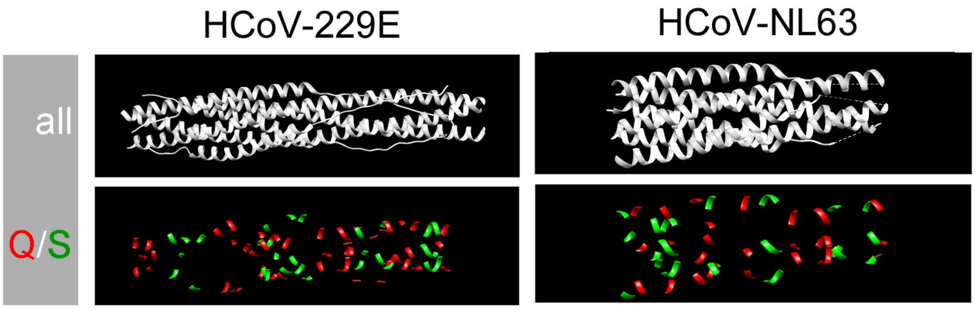

Suppl. Figure 2

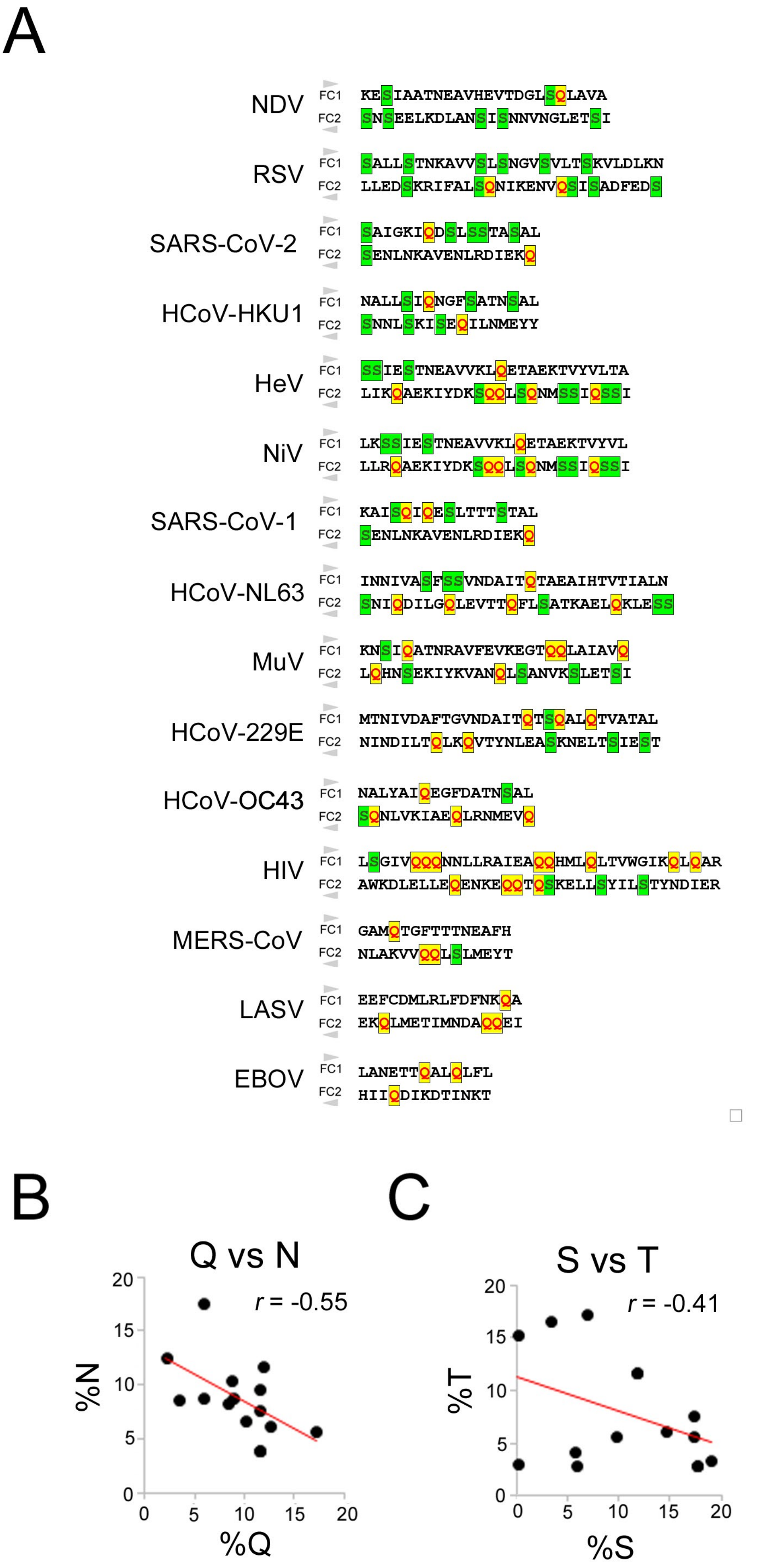

Suppl. Figure 4

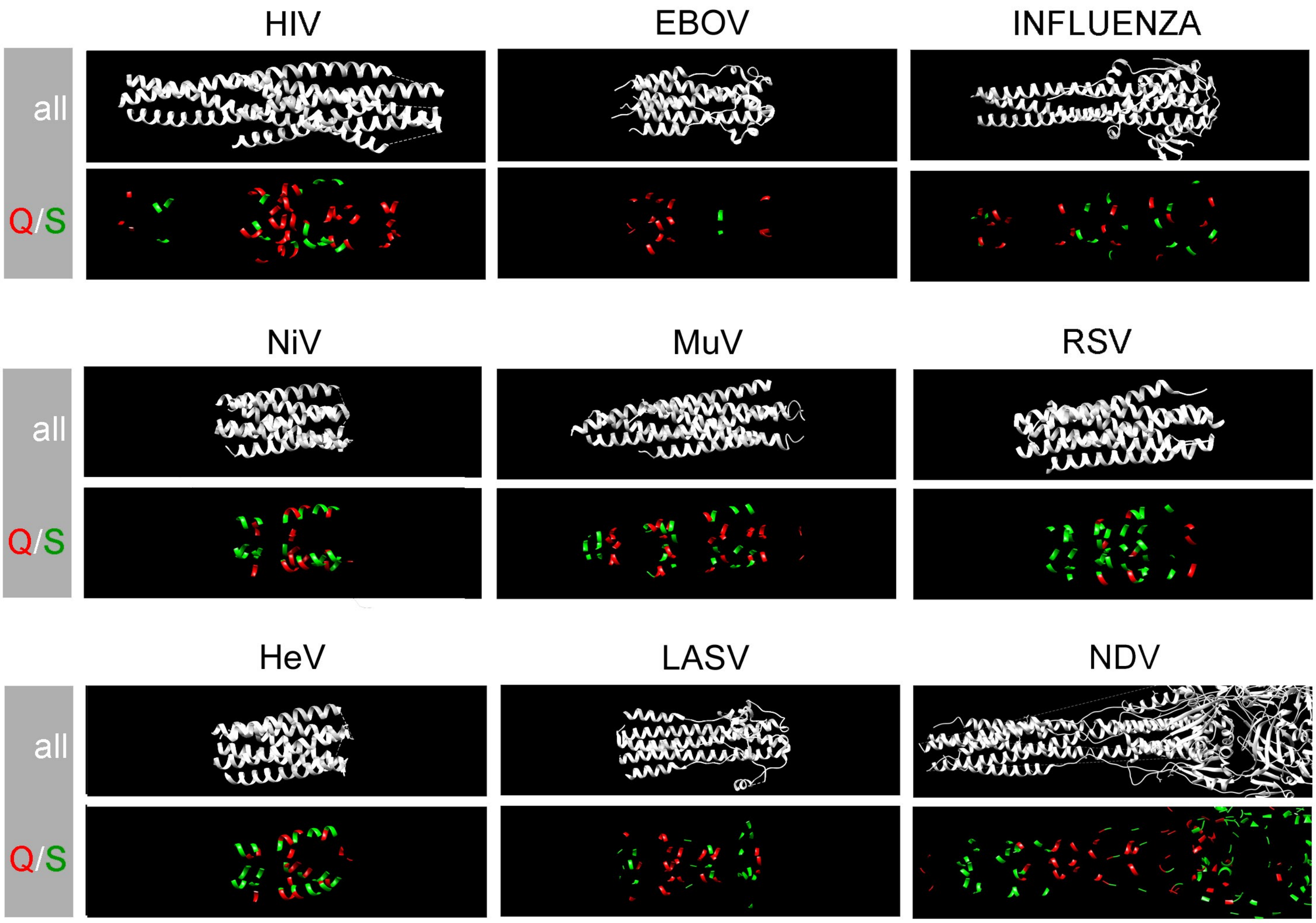

Suppl. Figure 3

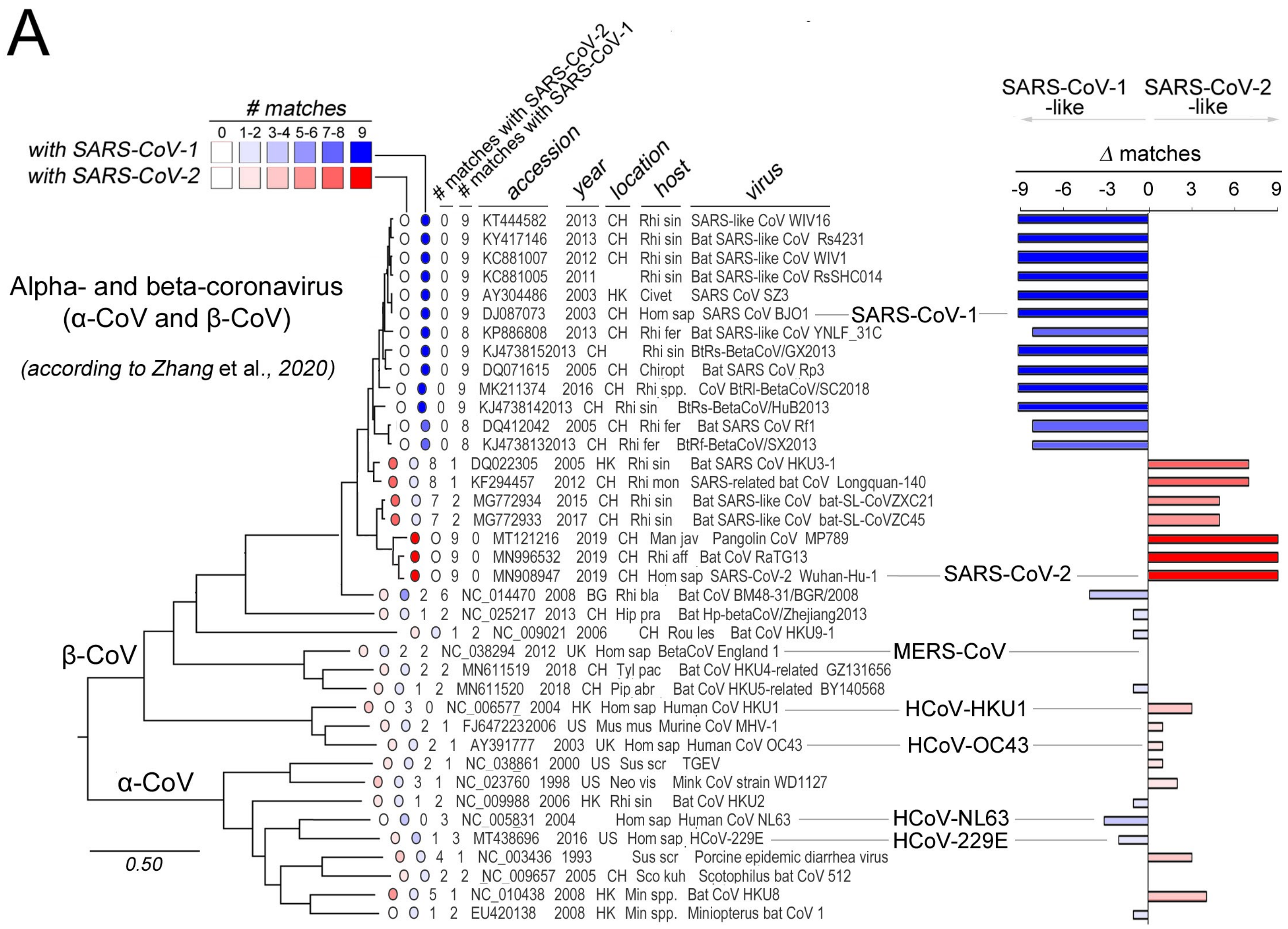

Suppl. Figure 5

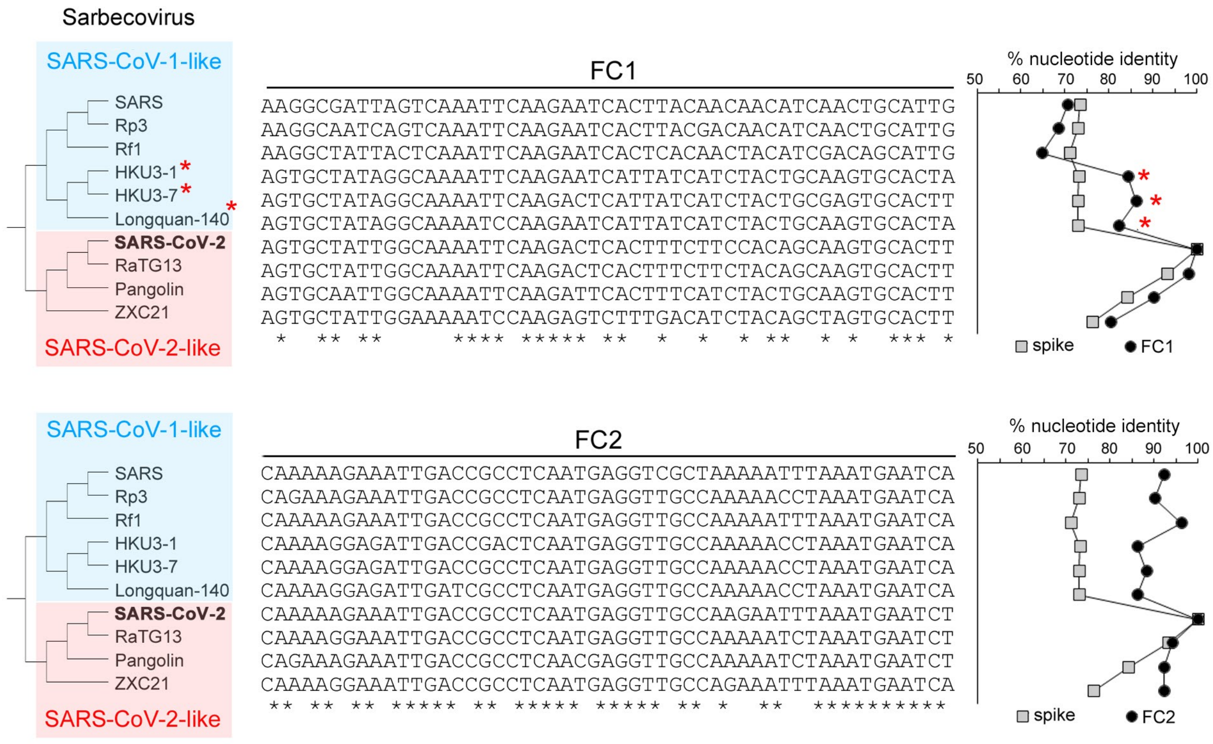

Suppl. Figure 6
